# Protective efficiency and immune responses to single and booster doses of formalin-inactivated scale drop disease virus (SDDV) vaccine in Asian seabass (*Lates calcarifer*)

**DOI:** 10.1186/s12917-024-04132-6

**Published:** 2024-06-20

**Authors:** Putita Chokmangmeepisarn, Saengchan Senapin, Suwimon Taengphu, Kim D. Thompson, Prapansak Srisapoome, Anurak Uchuwittayakul, Channarong Rodkhum

**Affiliations:** 1 Center of Excellence in Fish Infectious Diseases (CE FID), Faculty of Veterinary Science, Chulalongkorn University, Bangkok, 10330 Thailand; 2 Fish Health Platform, Center of Excellence for Shrimp Molecular Biology and Biotechnology (Centex Shrimp), Faculty of Science, Mahidol University, Bangkok, Thailand; 3 National Center for Genetic Engineering and Biotechnology (BIOTEC), National Science and Technology Development Agency (NSTDA), Khlong Nueng, Pathum Thani, Thailand; 4 Moredun Research Institute, Pentlands Science Park, Penicuik, EH26 0PZ UK; 5 Laboratory of Aquatic Animal Health Management, Department of Aquaculture, Faculty of Fisheries, Kasetsart University, Bangkok, 10900 Thailand; 6 Center of Excellence in Aquatic Animal Health Management, Faculty of Fisheries, Kasetsart University, Bangkok, 10900 Thailand

**Keywords:** Scale drop disease virus, SDDV, Formalin-inactivated vaccine, Asian seabass, Booster vaccination, Immune responses, Gene expression, IgM antibody

## Abstract

**Background:**

Scale drop disease virus (SDDV) threatens Asian seabass (*Lates calcarifer*) aquaculture production by causing scale drop disease (SDD) in Asian seabass. Research on the development of SDDV vaccines is missing an in-depth examination of long-term immunity and the immune reactions it provokes. This study investigated the long-term immune protection and responses elicited by an SDDV vaccine. The research evaluated the effectiveness of a formalin-inactivated SDDV vaccine (SDDV-FIV) using both prime and prime-booster vaccination strategies in Asian seabass. Three groups were used: control (unvaccinated), single-vaccination (prime only), and booster (prime and booster). SDDV-FIV was administered *via* intraperitoneal route, with a booster dose given 28 days post-initial vaccination.

**Results:**

The immune responses in vaccinated fish (single and booster groups) showed that SDDV-FIV triggered both SDDV-specific IgM and total IgM production. SDDV-specific IgM levels were evident until 28 days post-vaccination (dpv) in the single vaccination group, while an elevated antibody response was maintained in the booster group until 70 dpv. The expression of immune-related genes (*dcst, mhc2a1, cd4, ighm, cd8, il8, ifng*, and *mx*) in the head kidney and peripheral blood lymphocytes (PBLs) of vaccinated and challenged fish were significantly upregulated within 1–3 dpv and post-SDDV challenge. Fish were challenged with SDDV at 42 dpv (challenge 1) and 70 dpv (challenge 2). In the first challenge, the group that received booster vaccinations demonstrated notably higher survival rates than the control group (60% versus 20%, *P* < 0.05). However, in the second challenge, while there was an observable trend towards improved survival rates for the booster group compared to controls (42% versus 25%), these differences did not reach statistical significance (*P* > 0.05). These findings suggest that the SDDV-FIV vaccine effectively stimulates both humoral and cellular immune responses against SDDV. Booster vaccination enhances this response and improves survival rates up to 42 dpv.

**Conclusions:**

This research provides valuable insights into the development of efficient SDDV vaccines and aids in advancing strategies for immune modulation to enhance disease management in the aquaculture of Asian seabass.

## Background

Asian seabass (*Lates calcarifer*) is a commercially important aquaculture species, holding significant economic value within the Asia-Pacific region. In Thailand, it stands out as the most extensively cultured marine fish species averaging annual yields of 26,312 tons (constituting 86% of the total marine fish production) and generating an estimated value of 90 million USD annually [1]. Nevertheless, intensive fish culture has increased Asian seabass’s susceptibility to viral infections due to stressful, high-density conditions that weaken their immune systems and facilitate pathogen spread. Among these viral diseases, the megalocytiviruses, such as infectious and kidney necrosis virus (ISKNV), turbot reddish body iridovirus (TRBIV), red sea bream iridovirus (RSIV), and scale drop disease virus (SDDV), are highlighted as major contributors to economic losses and productivity challenges within the Asian seabass culture industry [2–6]. SDDV is a double-stranded DNA virus classified as a novel member of the genus *Megalocytivirus*, family *Iridoviridae* [2]. SDDV has been reported widely in southeast Asian countries as a causative agent of scale drop syndrome (SDS) in Asian seabass, resulting in high mortality in both natural and experimental infections [2, 7, 8]. Fish infected with SDDV typically exhibit clinical signs and pathological characteristics, which include darkened bodies, fin erosion, exophthalmia, scale loss, and hemorrhages [2, 7, 8].

Asian seabass cultivation requires a prolonged culture period, during which SDDV infection can pose a substantial threat [2, 8]. This underscores the need for an effective vaccination program to ensure protection throughout their growth cycle. Vaccination is one of the most successful measures for preventing the spread of various infectious diseases, including viral diseases. Inactivated vaccines were the earliest vaccine formulations to be tested due to their simple preparation, stability, safety, and cost-effectiveness [9]. However, their ability to induce immunity can be weaker than other vaccine types, potentially resulting in a shorter duration of protection [9]. This weaker immunogenicity may necessitate the requirement of adjuvants or booster vaccinations to obtain optimal immune stimulation and longer-term protection. Booster vaccination is a way to enhance the efficacy of vaccines, with studies demonstrating that the timing between the initial vaccination (prime) and the booster vaccination plays a crucial role in maximizing the vaccine’s effectiveness [10, 11]. Identifying immune markers can further guide the development of more effective vaccines by informing choices regarding protective antigens, vaccine formulations, antigen doses, and vaccination regimes [12]. While previous research has developed various vaccine formulations against SDDV infection in Asian seabass, including recombinant MCP, binary ethyleneimine, formalin-inactivated, and bivalent SDDV-ISKNV vaccines, these studies primarily focused on short-term protection (28 days post-vaccination) with limited exploration of long-term immunity and associated immune responses post-vaccination [2, 3].

The aim of this study was to investigate the efficacy of a prime or a prime-booster vaccination (at 28 days post-prime vaccination) with a formalin-inactivated SDDV vaccine administrated by intraperitoneal (IP) injection. The level of protection in the prime-booster vaccination group was compared with the prime vaccination group when the vaccinated fish were challenged with SDDV 6 and 10 weeks after the prime vaccination. Furthermore, to gain greater insight into the underlying immunological mechanisms, both serum and skin mucus IgM antibody levels were determined alongside the expression profiles of immune-related genes in both vaccinated and challenged fish. This approach aims to assess the long-term efficacy of the SDDV vaccine using a booster vaccination and elucidate the immunological processes triggered in Asian seabass by the vaccine.

## Methods

### Virus culture and vaccine preparation

The SDDV, strain SB11, was isolated from diseased Asian seabass during an SDS outbreak in Thailand [8]. Briefly, SDDV was propagated in a fish permission cell line and incubated at 27ºC for 5 days (unpublish data). The viral supernatant was collected and stored at -80ºC as a stock solution. This source of virus was used for both vaccine formulation and the challenge test. The concentration of the SDDV culture was quantified using a quantitative PCR (qPCR) for the *ATPase* gene, as previously described [13]. The SDDV stock solution (3.0 × 10^8^ DNA copies/mL) was inactivated with 0.037% formalin at 4ºC for 10 days [2]. Complete inactivation of the virus was verified by inoculating the formalin-inactivated SDDV vaccine (SDDV-FIV) onto the cell line. The development of a cytopathic effect (CPE) was monitored over 7 days using an inverted microscope. No CPE was observed in cultures inoculated with the inactivated virus.

### Experimental fish

Healthy Asian seabass (*Lates calcarifer*) weighting 15 ± 2 g were purchased from an Asian seabass hatchery farm of Amazon Group International Co., Ltd, Samut Sakhon, Thailand. The fish were acclimatized for seven days by maintaining in 500-L fiberglass tanks with 24 h of aerated water at a salinity of 10 ± 1 ppt, a temperature of 28 ± 1ºC, water pH of 7 ± 1 and dissolved oxygen (DO) of 5 ± 1 mg/L in tank water. The random selection of fish was screened to confirm they were free of iridoviruses by PCR, using OIE-approved primer sets [14], and bacterial screening was performed by conventional culture before performing the experiment. The fish were fed daily with a commercial carnivorous fish feed (protein 48%, fat 7%) diet (Profeed, Thai Union Feedmill Co., Ltd., Thailand) twice a day at 5% body weight.

### Experimental design

After being acclimatized to laboratory conditions, 750 Asian seabass were divided into three groups, consisting of control, single, and booster vaccination groups (*n* = 250/group). The control group was an unvaccinated group receiving only phosphate buffered saline (PBS); the single group received only one vaccination (prime vaccination); and the booster group received two vaccinations (prime and booster vaccinations) (*n* = 50, 5 replications). The vaccination schedule involved two doses administered at 0- and 28-days post vaccination (dpv). At 0 dpv, the fish in the single and booster groups were injected intraperitoneally (IP) with 0.1 mL of the SDDV-FIV (SDDV 10^7^ DNA copies/fish), while the control group was injected with 0.1 mL of sterile PBS. At 28 dpv, the fish in the control and single groups were injected with 0.1 mL of PBS, while the booster group was administered another dose of 0.1 mL of SDDV-FIV (SDDV 10^7^ DNA copies/fish). All fish were starved for 24 h and anesthetized with clove oil (20 ppm, Aqua-PEACE, Thailand) prior to vaccination.

### Sample collection

Serum and skin mucus were collected at seven-day intervals from 0 to 84 dpv (*n* = 5) to determine both SDDV-specific IgM antibody titers and total IgM antibody level. Skin mucus was collected by placing the fish in a sterile plastic bag containing 3 mL of 1× PBS and rubbing for 30 s. The collected skin mucus was centrifuged at 3,500 × g for 5 min, and the supernatants were kept at -20ºC until use. Blood was collected from the caudal vein and allowed to clot at 4ºC for 1 h. Then, serum was separated by centrifuging at 3,500 × g for 15 min and kept at -20ºC until use. Head kidney and peripheral blood lymphocytes (PBLs) were collected from each group (*n* = 4) at 0, 1, 3, 7, 28, 29, 31, 35, 42, 43, 45, 49, 70, 71, 73, and 77 dpv for subsequent analysis of immune-related gene expression by qRT-PCR. These time points represent 0, 1, 3, and 7 days after the prime vaccination (at 0 dpv), booster vaccination (at 28 dpv), challenge 1 (at 42 dpv), and challenge 2 (at 70 dpv).

One mL of blood was collected using an anticoagulant (EDTA, Honeywell, Germany). The sampled blood was used to isolate PBLs by density gradient centrifugation. Briefly, the whole blood was diluted with Roswell Park Memorial Institute (RPMI) 1640 medium (Gibco, UK) at a 1:2 volume ratio. The diluted blood was gently layered onto 3 mL of density gradient medium (Lymphoprep™, Stemcell Technologies, Oslo, Norway) and centrifuged at 400 × g for 30 min. The PBL fraction (white layer below the plasma layer) was collected and resuspended with RPMI. Viability and purity assessments of the isolated cells were conducted using trypan blue exclusion test to determine the percentage of viable cells and cell counting methods to ascertain the proportion of PBLs in relation to other cell types within the sample. Both PBLs and head kidney were preserved in 1 mL of TRIzol™ reagent (Thermo Fischer Scientific, USA) and stored at -80ºC until use.

### Growth performance

To determine the growth performance of the fish, 10 fish from each replicate (*n* = 50) were assessed, measuring their initial (Wi) and terminal (Wt) body weight (84 days) at the start and finish of the trial. Total weight gain (WG) and average daily growth rate (ADG) were calculated using the following formula: WG (g/84 days) = Wt -Wi, ADG (g/day) = (Wt -Wi)/t [15].

### Experimental challenge

A total of 60 fish from each group (12 fish/replicate) were transferred to new tanks for the SDDV challenge, which was performed at 42 and again at 70 dpv. Each fish was injected with 0.1 mL of SDDV (3.0 × 10^4^ DNA copies/mL) by IP injection. The fish of each group were subdivided into two separate tanks containing 30 fish for observing fish survival, while the remaining 30 were allocated for sample collection. The mortality rate and clinical signs were observed daily for 20 days. Relative percentage survival (RPS) was calculated using this formula: RPS (%) = [1 – (Mortality of vaccinated group/Mortality of control group)] × 100. The Kaplan Meier survival curves were generated using SPSS version 29.0.1. Tissue collection, including head kidney and PBLs, was collected from survival fish and conducted as described above for additional gene expression analysis. The samples were collected at two periods in the trial: 42, 43, 45, 49 dpv and 70, 71, 73, 77 dpv, corresponding to 0, 1, 3 and 7 days post-challenge (dpc) for challenges 1 and 2, respectively.

### IgM specific to SDDV analysis

The serum and skin mucus IgM levels specific to SDDV were determined using an indirect enzyme-linked immunosorbent assay (ELISA). Briefly, 96-well microplates were coated with 100 µL of SDDV-FIV (equivalent to SDDV 10^8^ DNA copies/mL) in coating buffer (0.01% poly-L-lysine, Elabscience®, USA) and incubated at 4ºC overnight. After blocking with blocking buffer (Visual Protein-BlockPRO™, Energenesis Biomedical, Taipei, Taiwan) and washing three times with PBST (PBS containing 0.05% Tween-20), 100 µL of diluted serum (1:200) and skin mucus (1:2) were added into the microplates and incubated at 4ºC overnight. Subsequently, the microplates were washed three times with PBST before being incubated with 100 µL of anti-Asian seabass IgM monoclonal antibody (diluted with PBS 1:33) (Marine Leader, Thailand) for 1 h at RT. Then, the microplates were washed three times with PBST and incubated with 100 µL of HRP-conjugated goat anti-mouse IgG (diluted with PBS 1:30,000) (Jackson Immuno Research Laboratories, USA) for 1 h at RT. After the last washing step, 100 µL of TMB One Component HRP Microwell Substrate (Surmodics, MN, USA) was added to the wells, and the reaction was allowed to develop at room temperature (RT) for 1 min in the dark. The reaction was stopped by adding 100 µL of TMB stop solution (Surmodics, MN, USA). Optical density (OD) was measured using a microplate reader at 450 nm (iMark™, Bio-Rad, USA). The OD of the negative reaction (no fish serum) was used as the cut-off value.

### Total IgM level analysis

The quantification of total IgM levels in serum and skin mucus was conducted using the same indirect ELISA protocol as specific IgM. Notably, the antigen coating step was altered by adding 100 µL of serum and skin mucus samples as coating agents on the wells. The following steps, including washing, antigen blocking, primary antibody anti-Asian seabass IgM monoclonal antibody [i.e. anti-Asian seabass IgM monoclonal antibody (Marine Leader, Thailand)], secondary antibody (i.e. HRP-conjugated goat anti-mouse IgG), substrate incubation, and stop reaction, were carried out for the SDDV-specific IgM detection. Optical density (OD) was measured using a microplate reader at 450 nm (iMark™, Bio-Rad, USA). The OD of the negative reaction (no fish serum) was used as the cut-off value.

### Quantitative real-time reverse transcriptase-PCR (qRT-PCR)

Head kidney (approximately 25 mg) and PBLs were preserved as mentioned above and subjected to RNA extraction using TRIzol™ reagent (Thermo Fischer Scientific, USA) according to the manufacturer’s protocol. The RNA concentration was quantified using a nano-spectrophotometer (NanoDrop™, Thermo Fischer Scientific, USA). A total of 1,000 ng of RNA was used as a template for synthesized cDNA according to the instructions of the Maxime™ RT PreMix Kit (Intron Biotechnology, Korea).

A total of nine immune-related gene expressions, including *lysozyme* (*lyz*), *myxovirus resistance* (*mx*), *interferon gamma* (*ifng*), *interleukin 8* (*il8*), *dendritic cell specific transcript* (*dcst*), *major histocompatibility complex class II integral membrane alpha chain gene* (*mhc2a1*), *CD4 molecule* (*cd4*), *CD8 molecule* (*cd8*), *immunoglobulin heavy constant mu* (*ighm*) were determined along with *beta actin (bact)* which served as a housekeeping gene for internal normalization. The qRT-PCR was carried out using the Mx3005P QPCR Systems instrument (Agilent, CA, USA). The qPCR reaction was carried out in a 20 µL reaction mixture containing 10 µL of 2× Brilliant III Ultra-Fast SYBR^®^ Green QPCR Master Mix (Agilent, CA, USA), 2 µL of 0.5 mM forward and reverse primers (Table 1), 2 µL of cDNA template, and 4 µL of DEPC-treated water (Thermo Fischer Scientific, USA). The qPCR condition was set as follows: initial denaturation at 95ºC; 40 cycles of 95ºC for 30 s, 55ºC for 30 s, and 72ºC for 90 s; and final extension at 72ºC for 10 min. The relative gene expression of the immune-related genes was calculated *via* the 2^−Δ∆CT^ method [16].

**Table 1:** Primers of immune-related genes used for RT-qPCR in this study

Primer name	Nucleotide sequence (5ˈ→ 3ˈ)	Gene	Amplicon size (bp)	T_m_ (˚C)	Reference
*Lc*_ *bact*-F	TACCCCATTGAGCACGGTATTG	*actin, beta* (*bact*)	160	60	[35]
*Lc*_ *bact* -R	TCTGGGTCATCTTCTCCCTGTT				
*Lc*_ *ighm* -F	TGTCAAGGTAAACGAGGGAGC	*immunoglobulin heavy constant mu* (*ighm*)	152	60	[35]
*Lc*_ *ighm* -R	TCCCCTGGATCCATTCGTCA				
*Lc*_*cd4-*F	AGTGCAATGGATTGGGGTAGATAA	*CD4 molecule* (*cd4*)	156	60	[36]
*Lc*_*cd4-*R	GTTGCAGGCTCTGTAACTTTGATT				
*Lc*_*mhc2a1*-F	TTCCTACCTCCCTGATCTACCC	*major histocompatibility complex class II integral membrane alpha chain gene* (*mhc2a1*)	178	60	[35]
*Lc*_*mhc2a1*-R	CTGAAGTCGCTGTTGGAGTAGT				
*Lc*_*cd8-*F	AATTCCTTCAGCAAAGCTCGTG	*CD8 molecule* (*cd8*)	188	60	XM_018700269.2
*Lc*_*cd8-*R	GTGGTTGTGGGTAGTCTTGGAT				
*Lc*_*dcst-*F	AAGACAGTAGACCTCTCCCACA	*dendritic cell specific transcript* (*dcst*)	170	60	[38]
*Lc*_*dcst-*R	CAAACAGGGGAAGGACTGAGAG				
*Lc*_*il8-*F	GCATCATCAAGGAGAGAAAGCC	*interleukin 8* (*il8*)	186	60	[37]
*Lc*_*il8-*R	GTGTCTGCTCAGCTTGTTTCTT				
*Lc*_*mx-*F	GAGGTCATCCACCTGAAGAAGG	*myxovirus resistance* (*mx*)	184	60	XM_051074677.1
*Lc*_*mx-*R	TGAGCTTCTCAGCCAGTTTAGG				
*Lc*_*ifng*-F	TACCAGGAGCAGGACAAGC	*interferon gamma* (*ifng*)	134	60	[39]
*Lc*_*ifng-R*	TCGTCAGGCAGCGAACTT				
*Lc_lys*-R	TGCATCACACACCATGGCAA	*lysozyme* (*lyz*)	401	60	[40]
*Lc_lys*-F	CATCCACGTTGTCATAGGAG				

### Statistical analysis

The homogeneity of variance for all collected data was assessed prior to conducting the ANOVA analysis. All data were presented as means ± SD, and all graphs were generated using GraphPad Prism 10. Statistical differences were analyzed by one-way ANOVA followed by Tukey’s post hoc test using SPSS version 29.0.1. The statistical differences were indicated as * (*P* < 0.05), ** (*P* < 0.01), and *** (*P* < 0.001).

## Results

### Growth performance parameters

The effect of the SDDV-FIV vaccination on the survival rate and growth performances of the vaccinated fish is shown in Table 2. Growth performance parameters based on body weight, including WG and ADG, were assessed at the end of the 84-day experiment. There were no significant differences in WG and ADG values among the groups. The weight gains of fish in the control, single, and booster groups were 14.98 ± 0.5 g, 14.8 ± 0.38 g, and 14.9 ± 0.44 g, respectively. The ADGs were 0.18 ± 0.008 g, 0.17 ± 0.006 g, and 0.17 ± 0.006 g, respectively (*P* > 0.05) (Table 2).

**Table 2:** Survival rate and growth performances of vaccinated and challenged Asian seabass

Group	Survival rate and RPS value								Growth performance	
	Vaccinated fish			Challenged fish					WG (g)	ADG (g)
	42 dpv	70 dpv		Challenge 1		Challenge 2				
	Survival rate (%)	Survival rate (%)		Survival rate (%)	RPS (%)	Survival rate (%)	RPS (%)			
Control	98.4%	98.4%		16.67^b^	-	40^b^	-		14.98 ± 0.57	0.18 ± 0.008
Single	97.2%	97.2%		33.33^b^	20	55^a^	25		14.8 ± 0.38	0.17 ± 0.006
Booster	98.0%	98.0%		66.67^a^	60	65^a^	42		14.9 ± 0.44	0.17 ± 0.006

Growth performance data are presented as mean ± SD (*n* = 50). Dash represents not available. Superscript letters in the same row indicate statistically significant differences (*P* < 0.05). RPS: relative percentage survival. WG: weight gain. ADG: average daily growth rate.

### SDDV-specific IgM levels

**Fig. 1:**
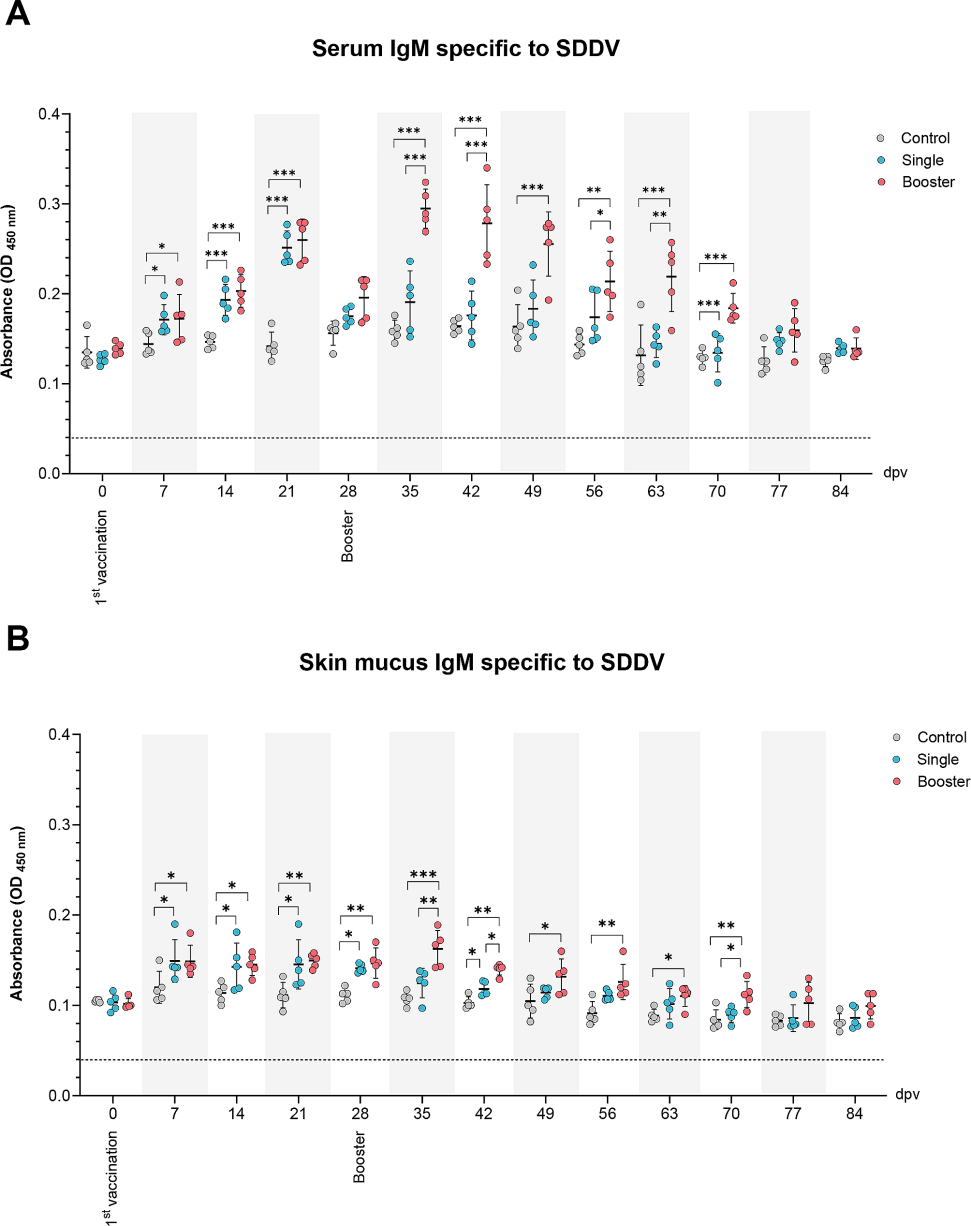
The levels of specific IgM against scale drop disease virus (SDDV) in the serum (**A**) and skin mucus (**B**) of vaccinated Asian seabass at intervals of 7 dpv using ELISA. Data are presented as mean ± SD (*n* = 5). Dash line indicates cut-off value of ELISA. Different asterisks indicate statistically significant differences between groups (*P < 0.05, **P < 0.01, ***P < 0.001)

The ELISA results of serum and skin mucus IgM levels against SDDV are shown in Fig. 1. In the vaccine groups, serum IgM began to elevate at 7 dpv and peaked at 21 dpv. The serum IgM levels of the vaccine groups at 7, 14, and 21 dpv were found to be significantly higher than those in the control group (*P* < 0.001, *P* < 0.01, *P* < 0.05). A decline in IgM levels was noted at 28 dpv in the vaccine groups. Following the booster dose (28 dpv), IgM levels in the booster group significantly increased, peaking at 35 dpv and persisting up to 70 dpv, with significantly higher levels compared to the control and single groups at 35, 42, 49, 56, 63, and 70 dpv (*P* < 0.001, *P* < 0.01, *P* < 0.05) (Fig. 1A).

For skin mucus specific IgM levels, the peak of IgM was noted at 7 dpv in both vaccine groups. Then, the IgM levels declined the following week but remained significantly higher than those in the control group from 7 to 28 dpv (*P* < 0.01, *P* < 0.05). After the booster dose, the IgM levels of the booster group peaked again at 35 dpv and remained significantly higher than the control group at 35, 42, 49, 56, 63, and 70 dpv (*P* < 0.001, *P* < 0.01, *P* < 0.05) (Fig. 1B).

### Total IgM levels

**Fig. 2:**
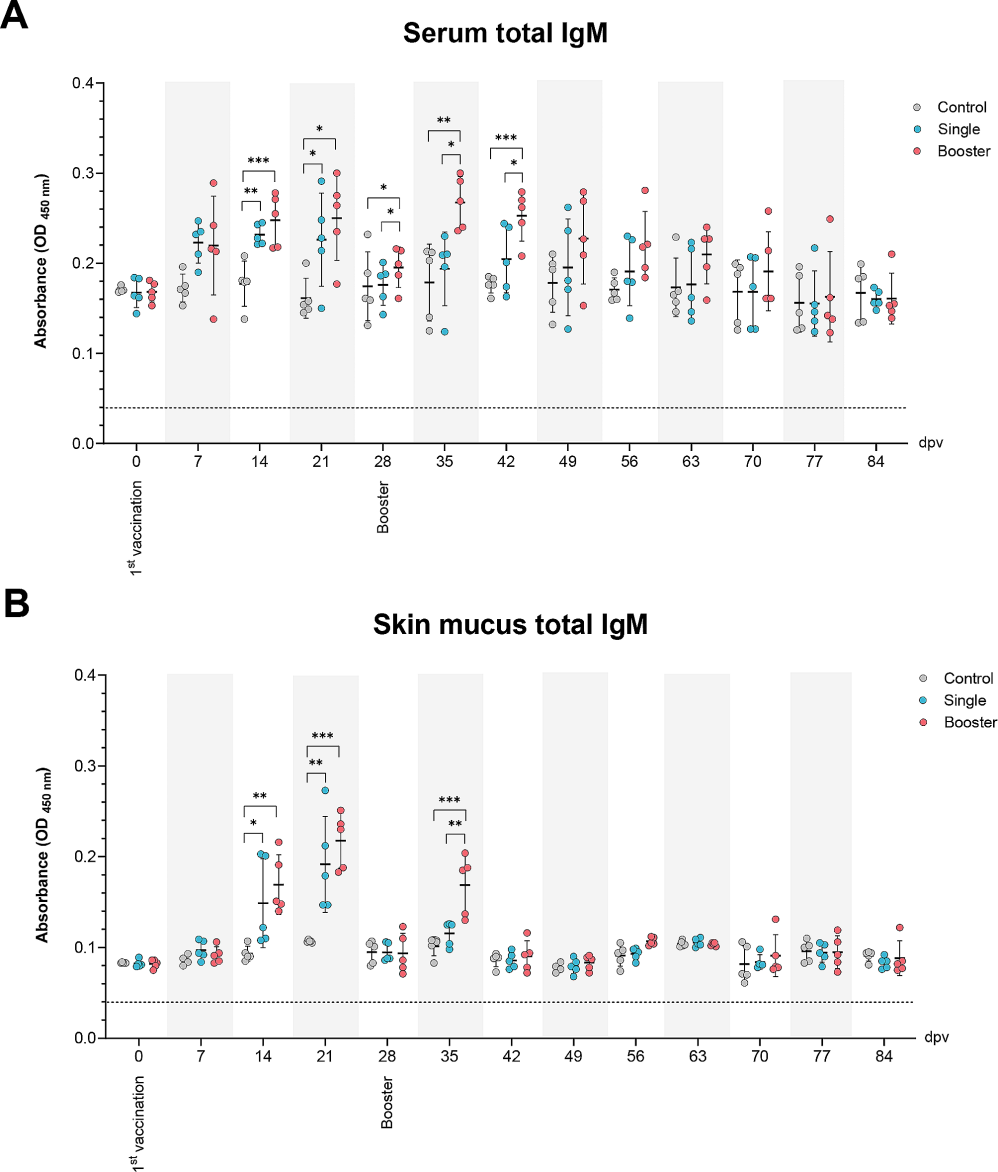
The levels of total IgM in the serum (**A**) and skin mucus (**B**) of vaccinated Asian seabass at intervals of 7 dpv using ELISA. Data are presented as mean ± SD (*n* = 5). Dash line indicates cut-off value of ELISA. Different asterisks indicate statistically significant differences between groups (*P < 0.05, **P < 0.01, ***P < 0.001)

The total serum and skin mucus IgM levels were measured weekly from 0 to 84 dpv using an indirect ELISA (Fig. 2). The total serum IgM levels were initially detected at 7 dpv and peaked at 21 dpv in the vaccine groups. Significantly higher IgM levels compared to the control group were observed at 14 and 21 dpv (*P* < 0.001, *P* < 0.01, *P* < 0.05). A decline in serum IgM levels was noted at 28 dpv in the vaccine groups, but the serum IgM levels of the booster group remained significantly higher than the single and control groups. After the booster vaccination (at 28 dpv), serum IgM levels in the booster group peaked again at 35 dpv and were significantly higher than the other groups at 35 and 42 dpv (*P* < 0.05, *P* < 0.01) (Fig. 2A).

Like the serum, skin mucus total IgM levels were obtained at 7 dpv and reached peak levels at 21 dpv in the vaccine groups. Significantly higher skin mucus IgM levels were observed in both vaccine groups compared to the control group at 14 and 21 dpv (*P* < 0.001, *P* < 0.01, *P* < 0.05). Then, the skin mucus IgM levels of the vaccine groups decreased at 28 dpv. After the booster vaccination, skin mucus IgM levels of the booster group peaked again at 35 dpv and then dropped at 56 dpv. The skin mucus IgM levels of the booster group were significantly higher than the other groups at 35 dpv (*P* < 0.001, *P* < 0.01) (Fig. 2B).

### Immune-related gene expression

Gene expression analysis of vaccinated fish.

Gene expression analysis was conducted using qRT-PCR to assess the expression of immune-related genes (*lyz*, *dcst, mhc2a1, cd4, ighm, cd8, il8, ifng*, and *mx*) in both head kidney and PBLs. Gene expression was evaluated in the vaccinated fish at multiple time points post-vaccination, including 0, 1, 3 and 7 days after prime and booster dose (28, 29, 31, 35 dpv). Additionally, gene expression was evaluated for an extended period of up to 77 dpv. Relative expression was calculated by comparing the vaccine groups (single and booster) to the control group.

**Fig. 3:**
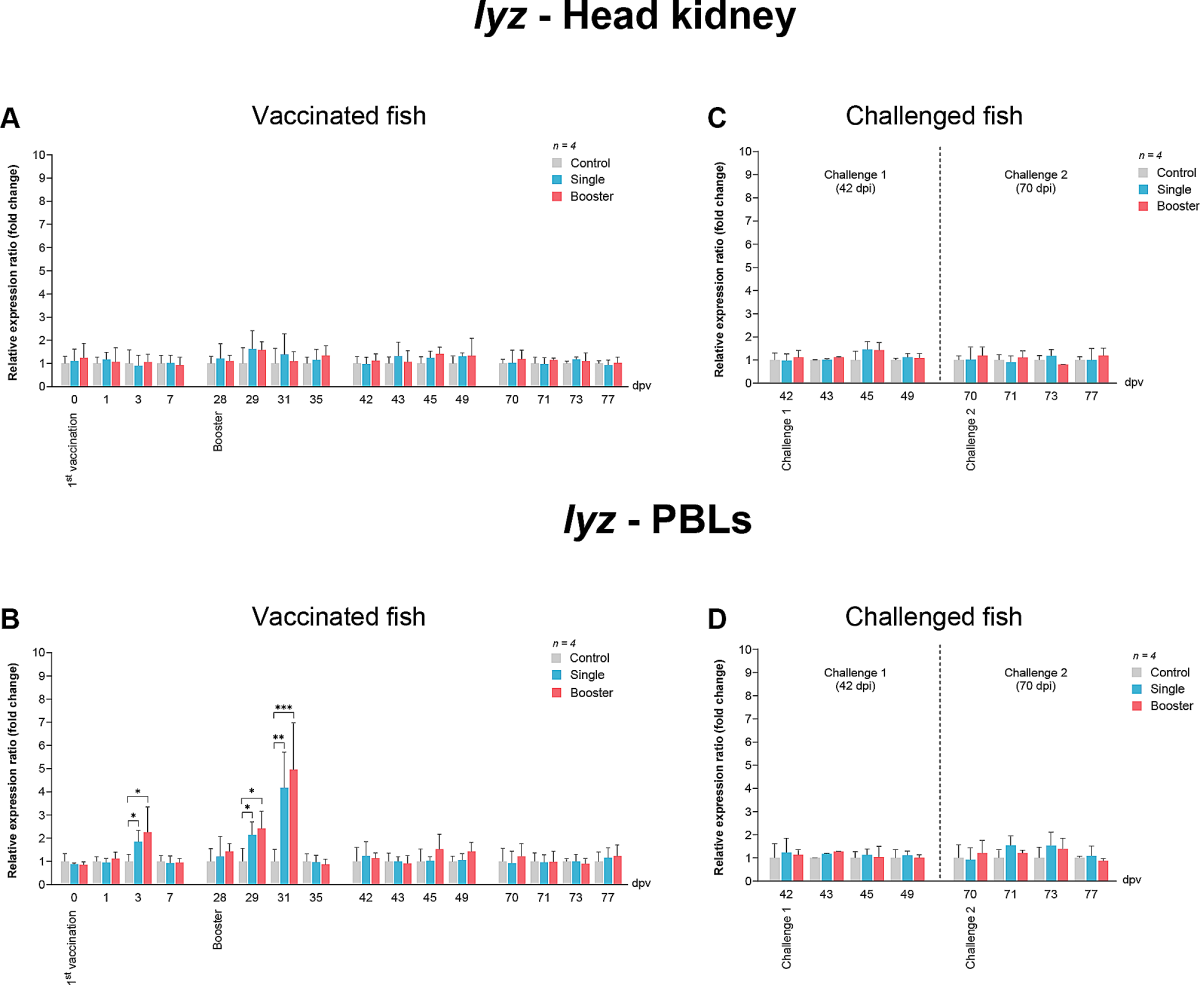
RT-qPCR analysis on the expression levels of *lyz* gene in vaccinated Asian seabass. The gene expression levels were normalized with *bact* and presented as fold-change to the expression of the control group. The bar graphs represent expression levels in head kidney (**A**) and PBLs (**B**) of vaccinated fish, and in head kidney (**C**) and PBLs (**D**) of vaccinated fish after challenge with SDDV. Data are presented as mean ± SD (*n* = 4). Different asterisks indicate statistically significant differences between groups (*P < 0.05, **P < 0.01, ***P < 0.001)

The expression level of *lyz* was not found to be significantly different in the head kidney between the groups of vaccinated fish (Fig. 3A). Conversely, the expression levels of *lyz* in PBLs of the vaccine groups were significantly higher than those of the control groups at 3, 29, and 31 dpv (*P* < 0.05, *P* < 0.01, *P* < 0.001) (Fig. 3B).

**Fig. 4:**
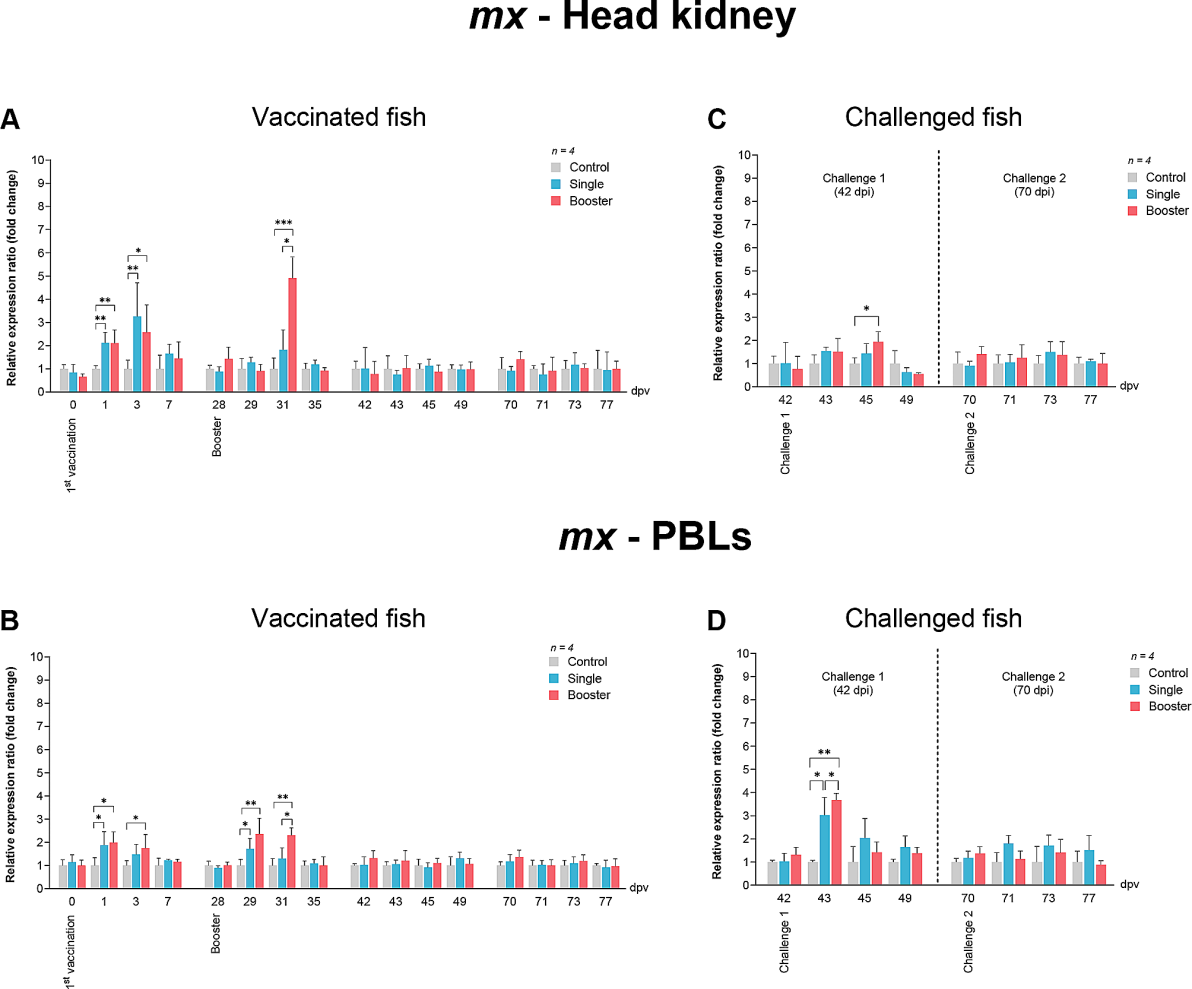
RT-qPCR analysis on the expression levels of *mx* gene in vaccinated Asian seabass. The gene expression levels were normalized with *bact* and presented as fold-change to the expression of the control group. The bar graphs represent expression levels in head kidney (**A**) and PBLs (**B**) of vaccinated fish, and in head kidney (**C**) and PBLs (**D**) of vaccinated fish after challenge with SDDV. Data are presented as mean ± SD (*n* = 4). Different asterisks indicate statistically significant differences between groups (*P < 0.05, **P < 0.01, ***P < 0.001)

In the head kidney, the expression level of *mx* was upregulated at 1 and 3 dpv in the vaccine groups, showing significantly higher levels compared to the control group (*P* < 0.05, *P* < 0.01) (Fig. 4A). After the booster vaccination, *mx* expression in the head kidney of the booster group significantly increased at 31 dpv compared to the single and control groups (*P* < 0.05, *P* < 0.001) (Fig. 4A). Likewise, *mx* expression in PBLs increased significantly at 1 dpv in the vaccine groups (*P* < 0.05) and at 3 dpv in the booster group (*P* < 0.05) (Fig. 4B). Furthermore, *mx* expression in PBLs was significantly upregulated again at 29 dpv in the vaccine groups, while in the booster group, it was significantly higher than in the single and control groups at 31 dpv (*P* < 0.05, *P* < 0.01) (Fig. 4B).

**Fig. 5:**
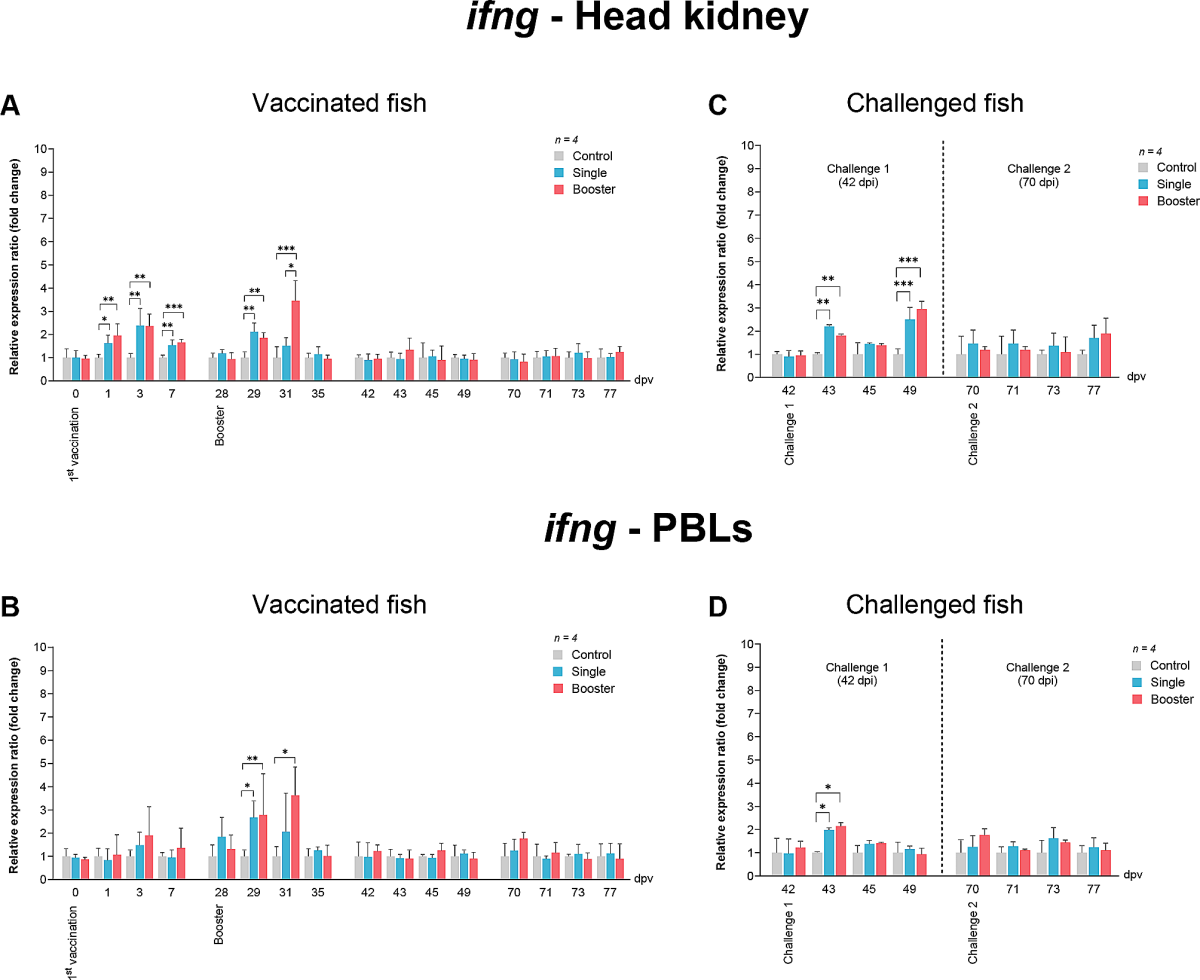
RT-qPCR analysis on the expression levels of *ifng* gene in vaccinated Asian seabass. The gene expression levels were normalized with *bact* and presented as fold-change to the expression of the control group. The bar graphs represent expression levels in head kidney (**A**) and PBLs (**B**) of vaccinated fish, and in head kidney (**C**) and PBLs (**D**) of vaccinated fish after challenge with SDDV. Data are presented as mean ± SD (*n* = 4). Different asterisks indicate statistically significant differences between groups (*P < 0.05, **P < 0.01, ***P < 0.001)

The expression levels of *ifng* in the head kidney of the vaccine groups were significantly higher than those of the control group at 1, 3, 7, 29, and 31 dpv (*P* < 0.05, *P* < 0.01, *P* < 0.001) (Fig. 5A). In PBLs, the expression levels of the vaccine groups were significantly elevated at 29 dpv (*P* < 0.01) (Fig. 5B). Moreover, at 31 dpv, the *ifng* expression level of the booster group was significantly higher than that of the other groups (*P* < 0.05, *P* < 0.001) (Fig. 5B).

**Fig. 6:**
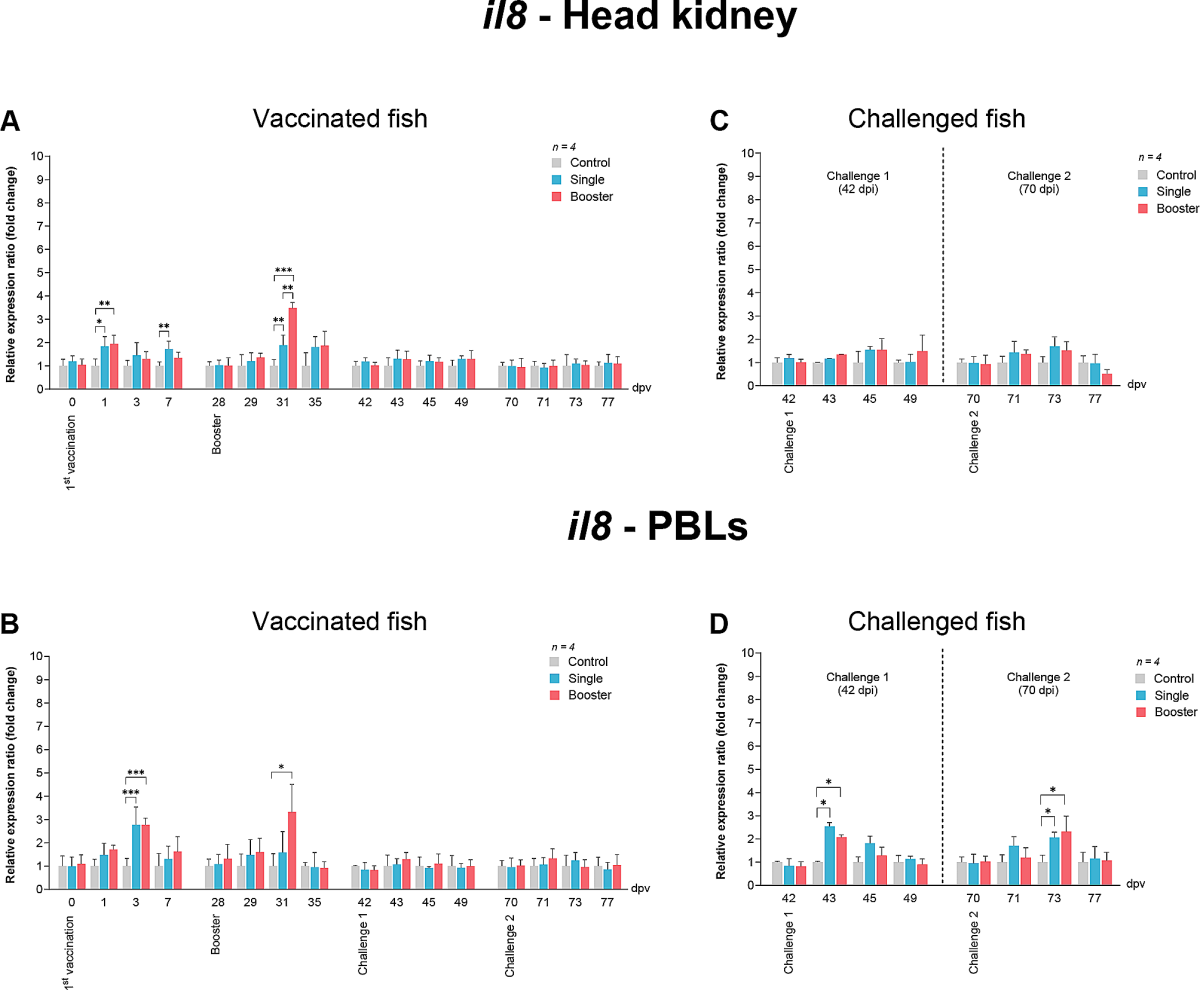
RT-qPCR analysis on the expression levels of *il8* gene in vaccinated Asian seabass. The gene expression levels were normalized with *bact* and presented as fold-change to the expression of the control group. The bar graphs represent expression levels in head kidney (**A**) and PBLs (**B**) of vaccinated fish, and in head kidney (**C**) and PBLs (**D**) of vaccinated fish after challenge with SDDV. Data are presented as mean ± SD (*n* = 4). Different asterisks indicate statistically significant differences between groups (*P < 0.05, **P < 0.01, ***P < 0.001)

For *il8* expression level in the head kidney (Fig. 6A the vaccine groups showed significantly higher *il8* levels at 1 dpv compared to the control group (*P* < 0.05, *P* < 0.01). At 7 dpv, the single group maintained significantly higher expression levels than both the control and booster groups (*P* < 0.01). Following the booster vaccination, the *il8* expression levels of the vaccine groups increased significantly at 31 dpv compared to the control group (*P* < 0.001, *P* < 0.01), and the expression level of the booster group was also significantly higher than that of the single group (*P* < 0.01). In PBLs (Fig. 6B), a significant increase was observed at 3 dpv in the vaccine groups compared to the control group. After the booster vaccination, only the booster group exhibited significantly higher expression levels at 31 dpv than the other groups (*P* < 0.05).

**Fig. 7:**
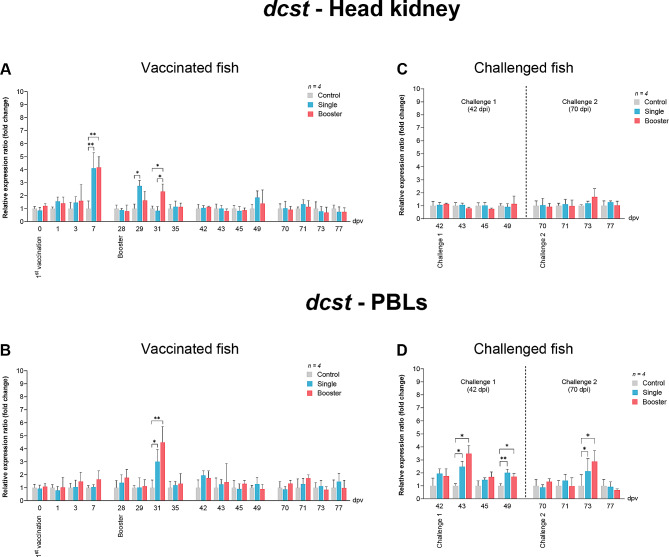
RT-qPCR analysis on the expression levels of *dcst* gene in vaccinated Asian seabass. The gene expression levels were normalized with *bact* and presented as fold-change to the expression of the control group. The bar graphs represent expression levels in head kidney (**A**) and PBLs (**B**) of vaccinated fish, and in head kidney (**C**) and PBLs (**D**) of vaccinated fish after challenge with SDDV. Data are presented as mean ± SD (*n* = 4). Different asterisks indicate statistically significant differences between groups (*P < 0.05, **P < 0.01)

The expression levels of *dcst* in the vaccine groups were significantly higher than those in the control group at 7 dpv (*P* < 0.01) (Fig. 7A). After the booster vaccination, the expression level of *dcst* in the head kidney of the single group significantly increased at 29 dpv compared to the control group (*P* < 0.05), while in the booster group, it was found to be significantly higher than in the control and single groups at 31 dpv (*P* < 0.05) (Fig. 7A). Meanwhile, in PBLs, significant differences in expression levels were noted at 31 dpv in the vaccine groups (*P* < 0.05, *P* < 0.01) compared to the control group (Fig. 7B).

**Fig. 8:**
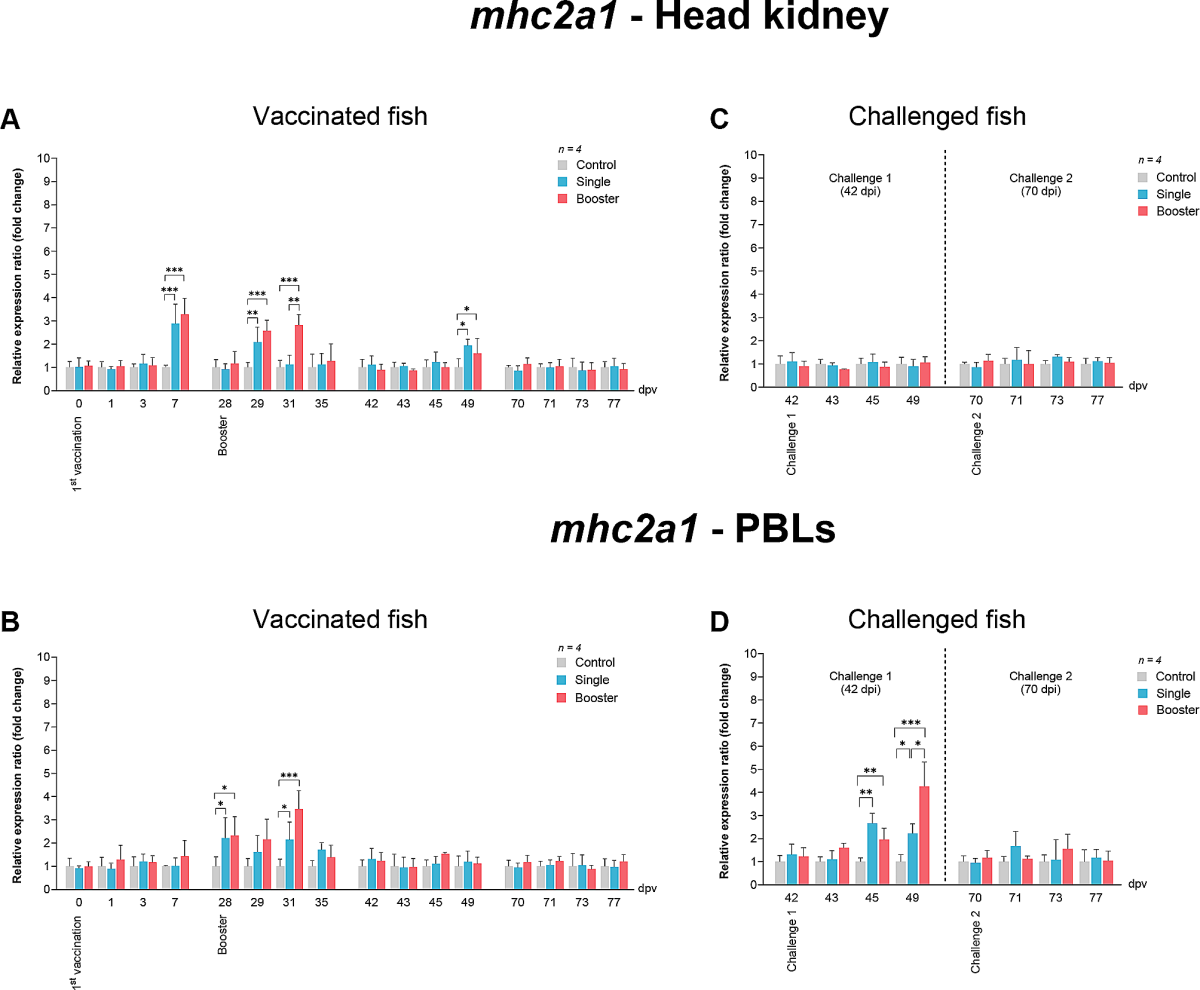
RT-qPCR analysis on the expression levels of *mhc2a1* gene in vaccinated Asian seabass. The gene expression levels were normalized with *bact* and presented as fold-change to the expression of the control group. The bar graphs represent expression levels in head kidney (**A**) and PBLs (**B**) of vaccinated fish, and in head kidney (**C**) and PBLs (**D**) of vaccinated fish after challenge with SDDV. Data are presented as mean ± SD (*n* = 4). Different asterisks indicate statistically significant differences between groups (*P < 0.05, **P < 0.01, ***P < 0.001)

In the head kidney, the expression levels of *mhc2a1* in the vaccine groups exhibited significant elevation at 7, 28, and 31 dpv (*P* < 0.001, *P* < 0.01), compared with the control group (Fig. 8A). The expression levels of *mhc2a1* were again increased at 49 dpv, and were significantly different to control levels (*P* < 0.05) (Fig. 8A). Concurrently, the expression levels of *mhc2a1* in PBLs were found to be upregulated after the booster vaccination, with significantly higher expression levels than those obtained for the control group at 28 and 31 dpv (*P* < 0.05, *P* < 0.001) (Fig. 8B).

**Fig. 9:**
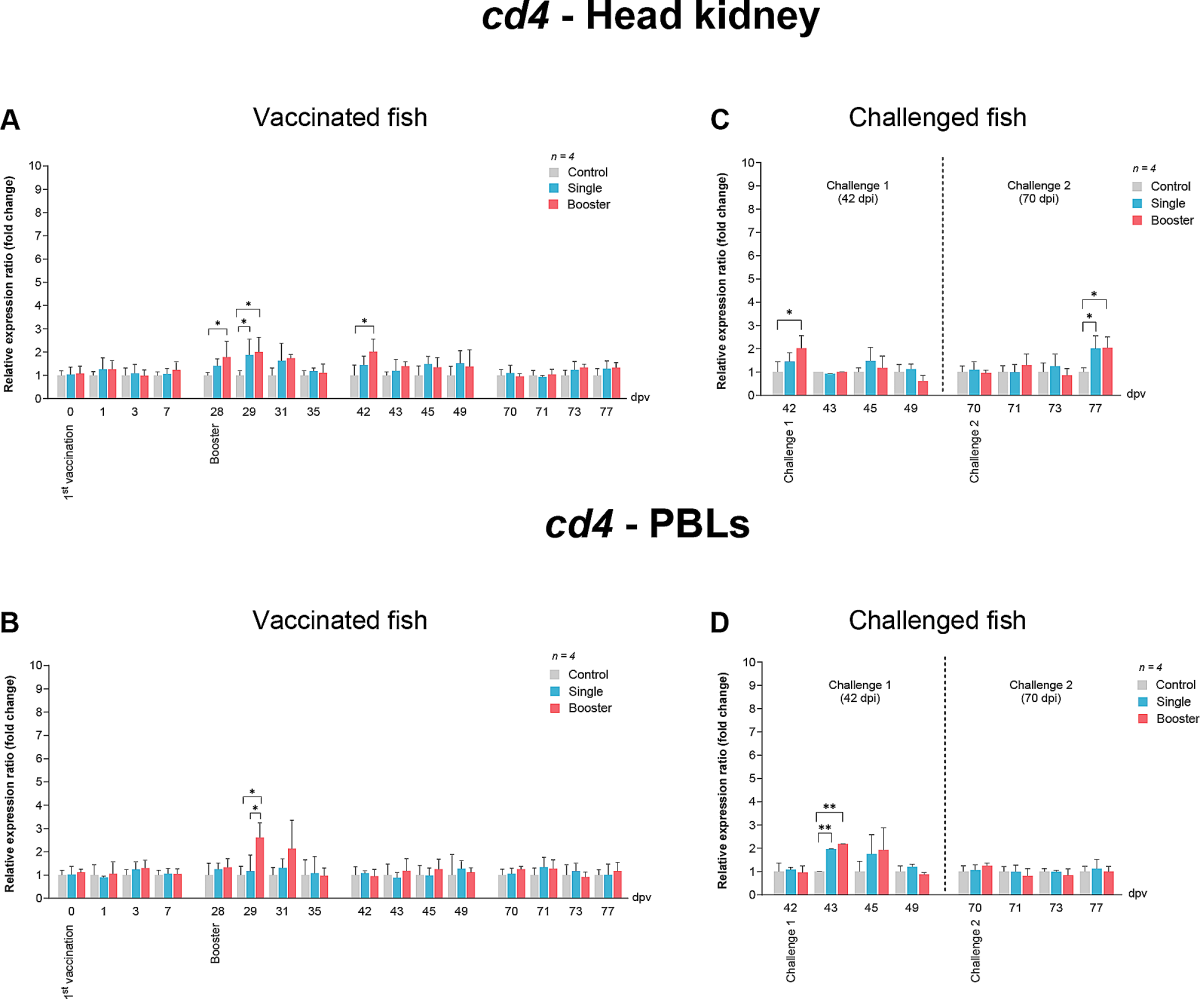
RT-qPCR analysis on the expression levels of *cd4* gene in vaccinated Asian seabass. The gene expression levels were normalized with *bact* and presented as fold-change to the expression of the control group. The bar graphs represent expression levels in head kidney (**A**) and PBLs (**B**) of vaccinated fish, and in head kidney (**C**) and PBLs (**D**) of vaccinated fish after challenge with SDDV. Data are presented as mean ± SD (*n* = 4). Different asterisks indicate statistically significant differences between groups (*P < 0.05, **P < 0.01)

As shown in Fig. 9A, the expression levels of *cd4* in the head kidney of the booster group were significantly increased at 28 dpv compared to the control group (*P* < 0.05). Subsequently, at 29 dpv, the vaccine groups exhibited significantly higher *cd4* expression than the control group (*P* < 0.05). Notably, by 42 dpv, the booster group maintained significantly elevated levels of expression compared to both the single and control groups (*P* < 0.05). Meanwhile, in PBLs, the *cd4* expression levels of the vaccine groups were significantly higher than those of the control group at 29 dpv (*P* < 0.05) (Fig. 9B).

**Fig. 10:**
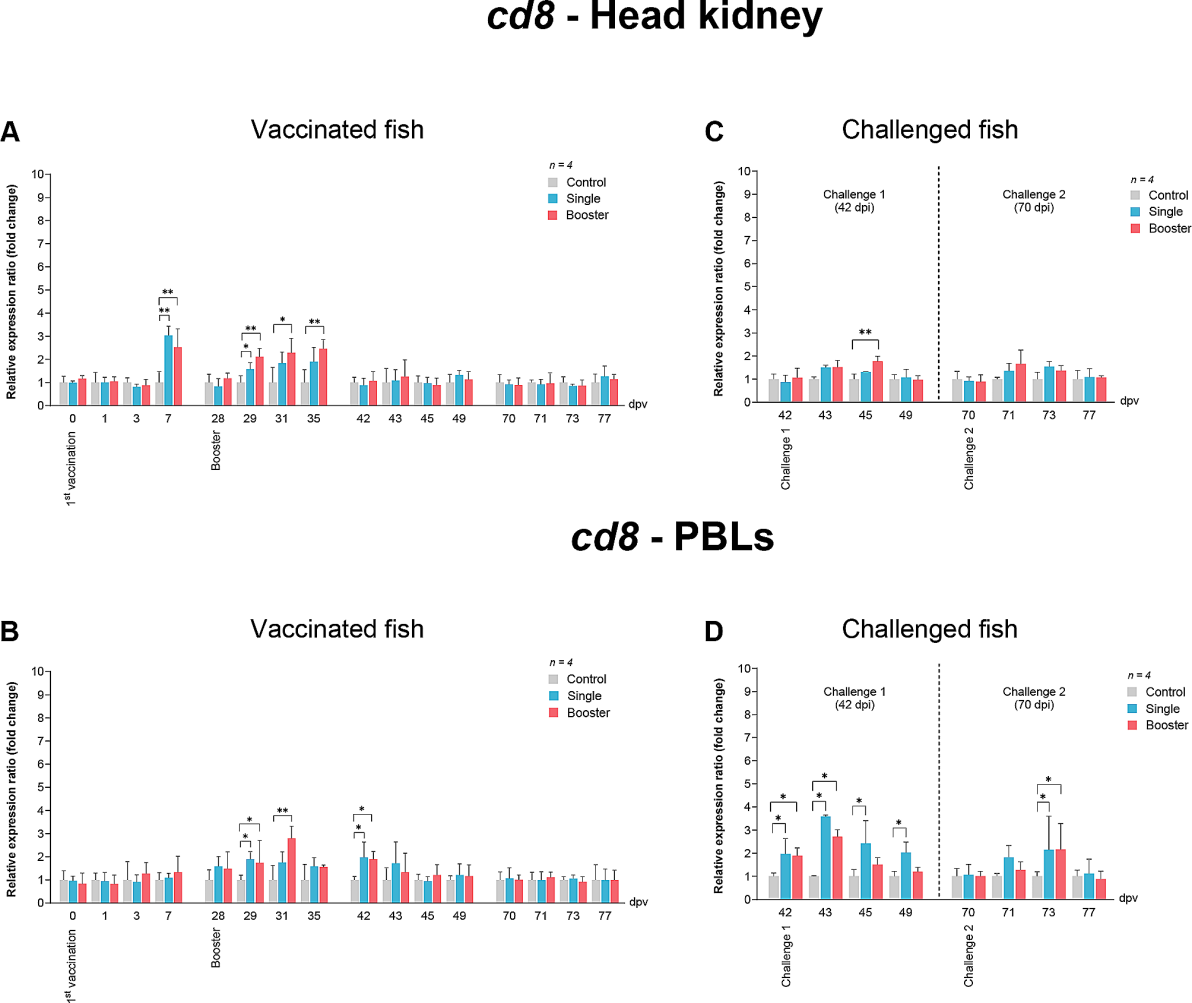
RT-qPCR analysis on the expression levels of *cd8* gene in vaccinated Asian seabass. The gene expression levels were normalized with *bact* and presented as fold-change to the expression of the control group. The bar graphs represent expression levels in head kidney (**A**) and PBLs (**B**) of vaccinated fish, and in head kidney (**C**) and PBLs (**D**) of vaccinated fish after challenge with SDDV. Data are presented as mean ± SD (*n* = 4). Different asterisks indicate statistically significant differences between groups (*P < 0.05, **P < 0.01)

For *cd8* expression, the vaccine groups showed significant upregulation at 7 and 29 dpv compared to the control group (*P* < 0.01). Notably, the booster group had significantly higher expression levels than the other groups at 31 and 35 dpv (*P* < 0.05, *P* < 0.01) (Fig. 10A). In PBLs, there was a significant increase at 29 dpv in the vaccine groups compared to the control group (*P* < 0.05). In comparison, at 31 dpv, the booster group retained significantly higher expression levels compared to the control group (*P* < 0.01) (Fig. 10B). At 42 dpv, the expression levels of the vaccine groups were upregulated again and significantly higher than the control (*P* < 0.05) (Fig. 10B).

**Fig. 11:**
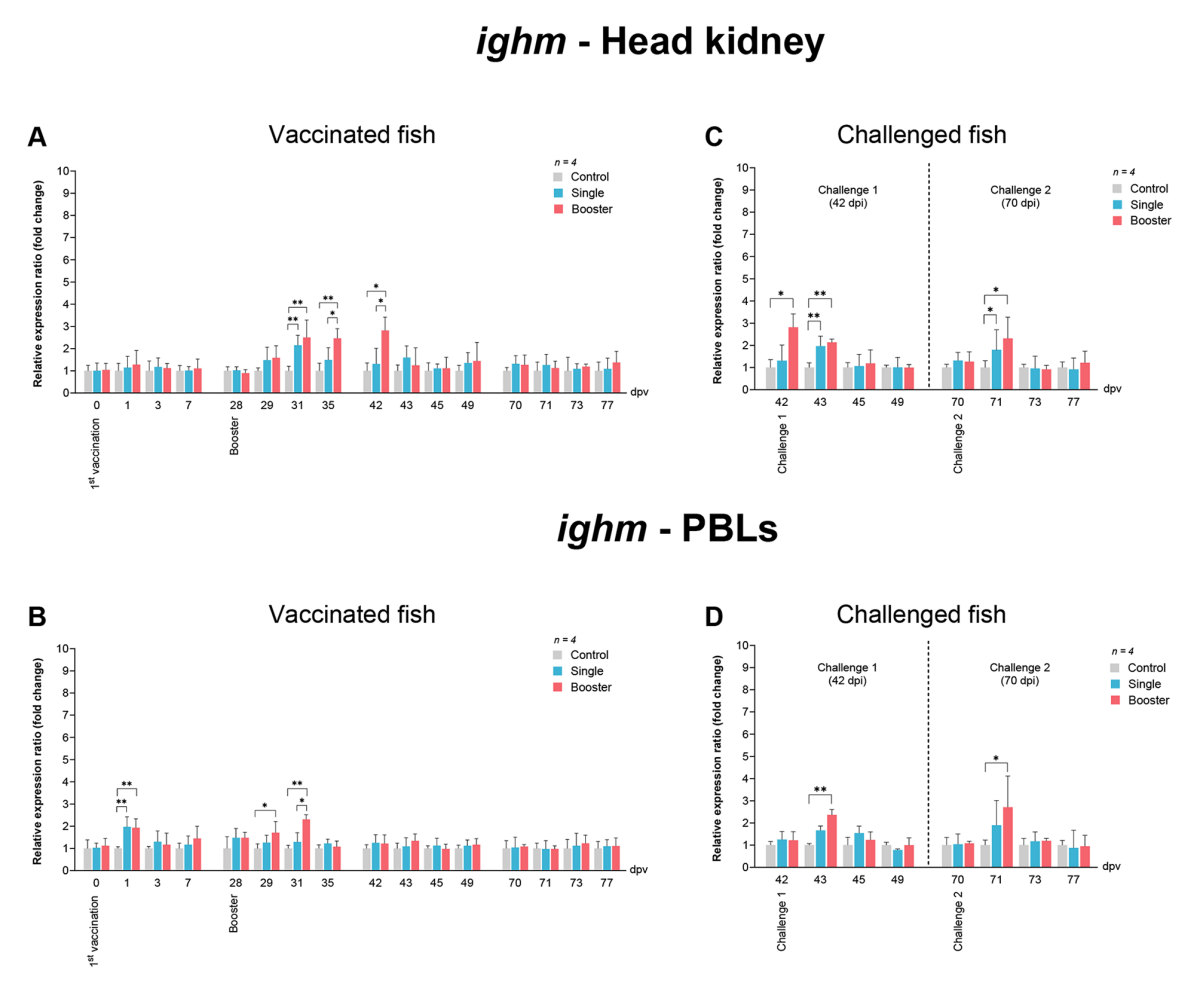
RT-qPCR analysis on the expression levels of *IgM* gene in vaccinated Asian seabass. The gene expression levels were normalized with *β-actin* and presented as fold-change to the expression of the control group. The bar graphs represent expression levels in head kidney (**A**) and PBLs (**B**) of vaccinated fish, and in head kidney (**C**) and PBLs (**D**) of vaccinated fish after challenge with SDDV. Data are presented as mean ± SD (*n* = 4). Different asterisks indicate statistically significant differences between groups (*P < 0.05, **P < 0.01)

In terms of *ighm* expression, a significant increase was observed in the head kidney of the vaccine groups at 31 dpv (*P* < 0.001, *P* < 0.01) compared to the control group, while the booster group had significantly higher expression levels compared to both the single and control groups at 35 and 42 dpv (*P* < 0.05, *P* < 0.01) (Fig. 11A). In PBLs, a notable rise in *ighm* expression was observed at 1 dpv in the vaccine groups compared to the control group (*P* < 0.01) (Fig. 11B). Furthermore, at 29 dpv, the booster group exhibited significantly higher expression levels compared to the control group (*P* < 0.05), and at 31 dpv, it exhibited significantly higher expression levels compared to both the single and control groups (*P* < 0.05, *P* < 0.01) (Fig. 11B).

**Fig. 12:**
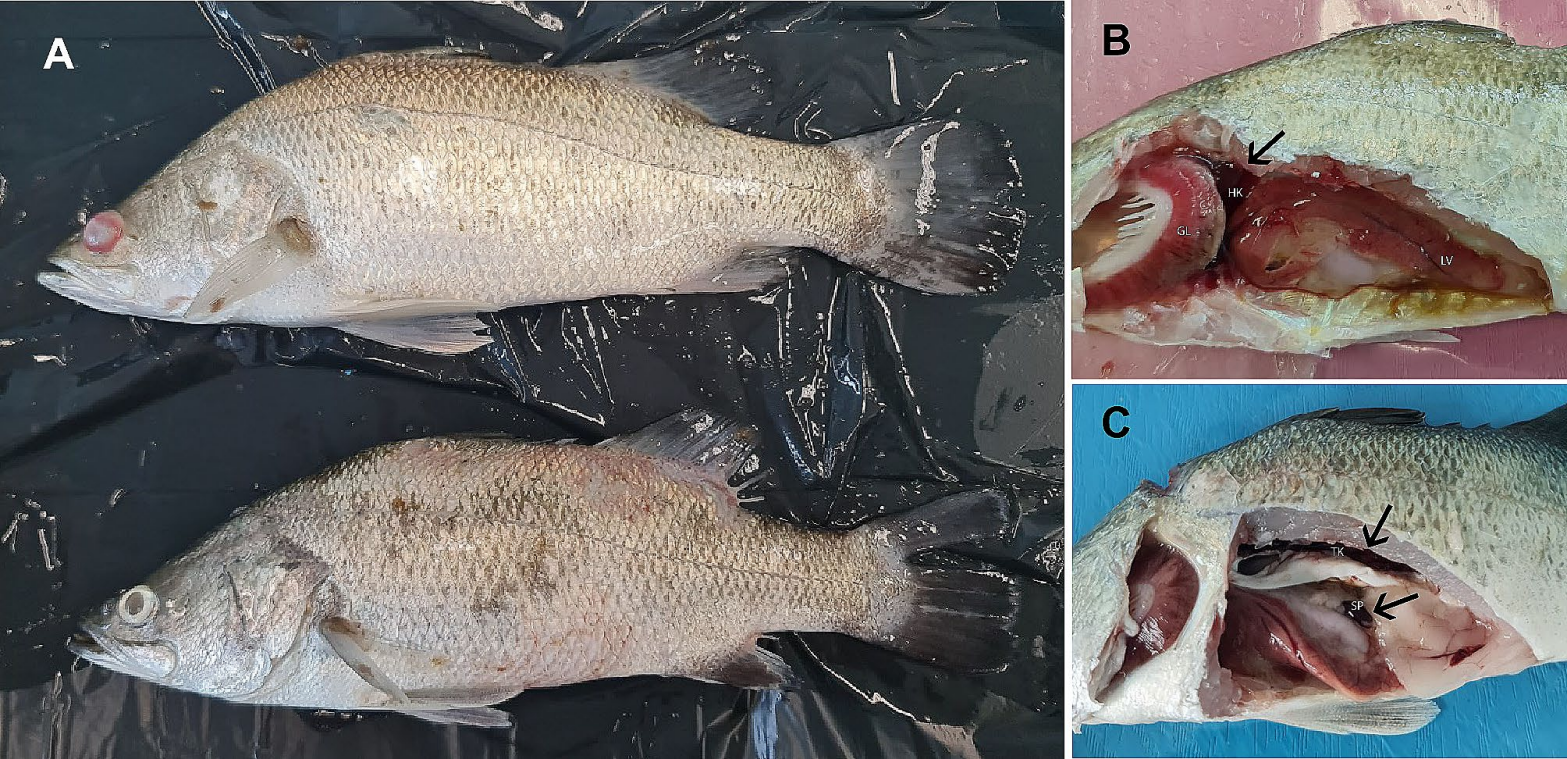
Gross lesions of Asian seabass challenged with scale drop disease virus (SDDV). External lesions revealed cloudy eye, fin and tail erosion, skin hemorrhage, detached scale and severe scale loss (**A**). Internal lesions revealed severe liver hemorrhage, enlarged major organs (black arrow) including spleen, head and trunk kidney (**B**-**C**). GL: gill, HK: head kidney, LV: liver, SP: spleen, TK: trunk kidney

### Gene expression analysis of challenged fish

For the challenged fish, which were vaccinated and infected with SDDV, gene expression analysis was carried out at 0, 1, 3, and 7 days after challenge 1 (at 42 dpv) and challenge 2 (at 70 dpv). Relative expression was calculated by comparing the vaccine groups (single and booster) to the control group.

The *lyz* expression levels in both head kidney and PBLs did not significantly increase in any groups after challenges 1 and 2 (Fig. 3C and D).

There was a significantly elevated expression of *mx* in the head kidney of the booster group compared to the control at 45 dpv (Fig. 4C). In PBLs, significant upregulation was observed in the vaccine groups at 43 dpv compared to the control group (*P* < 0.05) (Fig. 4D).

In the head kidney, *ifng* expression levels of the vaccine groups were significantly higher than the control group at 43 and 49 dpv (*P* < 0.001, *P* < 0.01) (Fig. 5C), while in PBLs, the significantly higher expression level was noted in the vaccine groups compared to the control group (*P* < 0.05) (Fig. 5D).

The *il8* expression levels in the head kidney did not show any significant difference between groups (Fig. 6C However, significantly higher levels of *il8* expression were found in PBLs of the vaccine groups at 43 dpv compared to the control group (*P* < 0.05). For challenge 2, significantly increased *il8* levels were observed at 73 dpv in PBLs of the vaccine groups compared to the control group (*P* < 0.05) (Fig. 6D).

There were no significant changes in *DC* expression in the head kidney across all groups (Fig. 7C). In PBLs, significantly higher levels were observed in the vaccine groups at 43 and 49 dpv in challenge 1 and at 73 dpv in challenge 2 compared to the control group (Fig. 7D) (*P* < 0.01, *P* < 0.05).

The *mhc2a1* expression in the head kidney did not show significant alterations in all groups (Fig. 8C). In contrast, significantly higher expression levels were noted in PBLs of the vaccine groups at 45 and 49 dpv (*P* < 0.01, *P* < 0.05) (Fig. 8D). Moreover, at 49 dpv, the booster group exhibited significantly higher expression levels than the single group (*P* < 0.05).

The expression levels of *cd4* in the head kidney of the booster group were significantly higher than both the single and control group (*P* < 0.05) (Fig. 9C). After challenge 2, upregulation of *cd4* was observed at 77 dpv in the vaccine groups (*P* < 0.05) (Fig. 9C). Meanwhile, in PBLs, the vaccine groups exhibited significantly higher levels compared to the control groups (*P* < 0.01) (Fig. 9D).

Regarding the expression levels of *cd8* in the head kidney of the booster groups significantly increased at 45 dpv compared to the control group (*P* < 0.01) (Fig. 10C). In PBLs, the expression levels of the vaccine groups were significantly upregulated at 42 and 43 dpv, whereas only the single group maintained significantly higher levels than the control group at 45 and 49 dpv (*P* < 0.05) (Fig. 10D). Moreover, the significant upregulation was noted at 73 dpv in PBLs of the vaccine groups (*P* < 0.05) (Fig. 10D).

Lastly, for *ighm* expression levels in the head kidney, the booster groups showed significantly higher expression levels than both the single and control groups at 42 dpv (*P* < 0.05) (Fig. 11C). Subsequently, at 43 dpv (challenge 1) and 71 dpv (challenge 2), the vaccine groups showed significantly higher expression levels compared with the control group (*P* < 0.01) (Fig. 11D). Similarly, in PBLs, the expression levels of the booster groups were significantly increased at 43 dpv and 71 dpv, compared to both the single and control groups (*P* < 0.01) (Fig. 11D).

### Survival rate of vaccinated Asian seabass

**Fig. 13:**
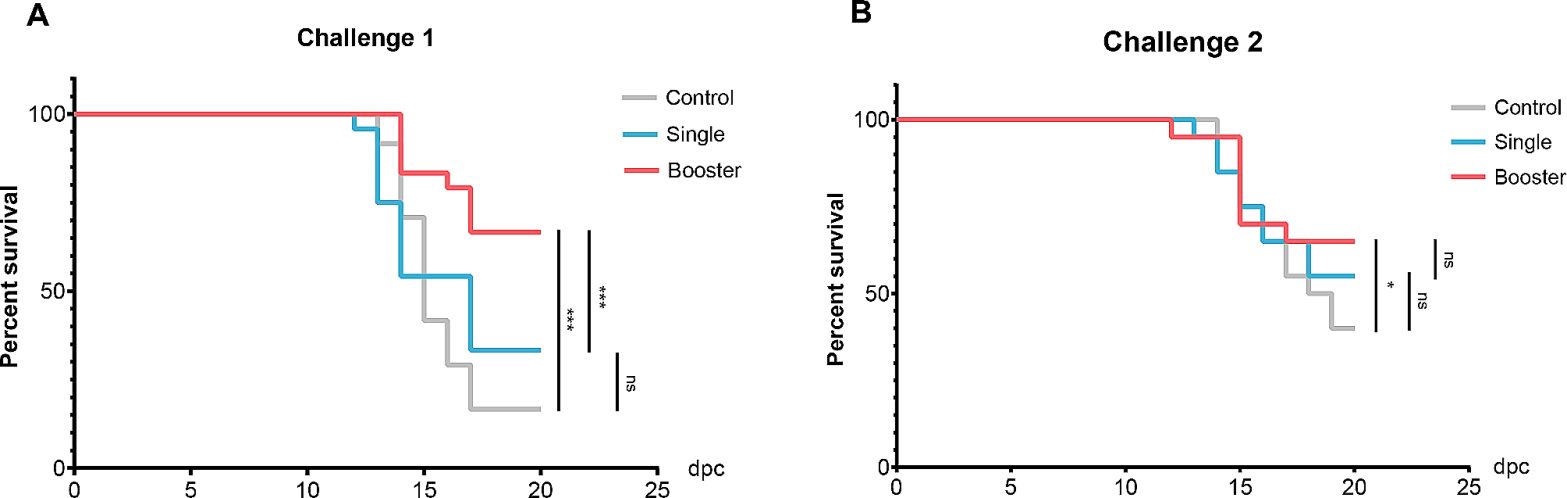
Kaplan-Meier survival curves showing the percentage survival of vaccinated Asian seabass after challenge with scale drop disease virus (SDDV) at 42 dpv (Challenge 1) (**A**) and 70 dpv (Challenge 2) (**B**). Different asterisks indicate statistically significant differences between groups (ns, not significant, * *P* < 0.05, *** *P* < 0.001)

The protective efficacy of the FKV was evaluated through two independent challenge tests (challenges 1 and 2) (Table 2). In challenge 1, conducted at 42 dpv, the onset of mortality was observed at 13 dpc, with mortality peaking at 14 dpc. The survival rates of the control, single, and booster groups were 16.67%, 33.33%, and 66.67%, respectively, while the calculated RPS value of the single and the booster group were 20% and 60%, respectively, which were significantly higher than the control group (*P* < 0.001) (Fig. 13A). In challenge 2, conducted at 70 dpv, the onset of mortality was observed at 12 dpc while the mortality was highest at 15 dpc. The survival rates of the control, single, and booster groups were 40%, 55%, and 65%, respectively, while the calculated RPS of single and booster groups were 25% and 42%, respectively. The RPS of the booster group was significantly higher than the control group (*P* < 0.05), while the RPS between the single and control group were not significantly different (Fig. 13B). Clinical signs of the SDDV infection, including darkening of the body, fin and tail erosion, and cloudy eyes with reddish circle were initially observed within 7 dpc in both challenge 1 and 2. Moreover, when moribund fish were necropsied, gross lesions were evident, together with skin hemorrhaging, loose scales, pale gills, severe hemorrhaging in the liver, splenomegaly, and swelling of both the head and the trunk kidney (Fig. 12).

## Discussions

SDDV is a significant virus pathogen in marine fish, causing scale drop disease (SDD) in certain fish species, including Asian seabass and yellow seabream. Asian seabass is recognized as particularly vulnerable to SDDV infections, leading to considerable economic losses [2, 17]. Consequently, there is a growing interest in developing preventive measures to control the disease, such as vaccination. Our study aimed to assess the effectiveness of a formalin-inactivated SDDV vaccine, together with a booster vaccination regime, and to assess the fish’s immune responses to the vaccine. This knowledge would assist in creating a vaccination plan for Asian seabass in aquaculture, aimed at developing a vaccine against SDDV to safeguard the fish throughout their entire production cycle.

The antibody response to a vaccine, particularly IgM responses, can indicate immune protection in fish [12]. The suggestion that higher antibody levels correlate with improved protection after exposure suggests that identifying a protective antibody level could be a method to assess the effectiveness of newly developed vaccines, particularly for the SDDV vaccine, for which no immunological data related to vaccination exists [18–21]. In this study, vaccinated fish showed a significant increase in specific IgM levels in both serum and skin mucus. This increase peaked at 42 dpv for those receiving a single dose and at 70 dpv for those who received a booster dose, in contrast to the control group. The pattern of IgM levels observed was directly associated with survival rates. The group that received the booster maintained elevated IgM levels in both serum and skin mucus up to the initial challenge at 42 dpv, resulting in significantly higher survival rates than both the control and single-dose groups. However, by the time of the second challenge at 70 dpv, these IgM levels had diminished, aligning with a reduced survival rate relative to the control group. These findings suggest that specific IgM plays a crucial role in establishing protection against SDDV infection.

Comprehending the dynamics of immune gene expression in response to vaccination and infection helps reveal how vaccines steer the host’s immune response towards eliminating pathogens [18]. The expression of immune genes that represent both innate (*lyz*, *dcst, cd4, ighm, cd8, il8, ifng*, and *mx*) and adaptive immunity (*mhc2a1, cd4*, *cd8*, and *ighm*) were determined in the recent study. Most of the innate immunity genes displayed rapid expression within 1–7 dpv, while those associated with adaptive immunity showed expression at a later stage, after 28 dpv. This finding reflects the basis of the immune system; innate immunity offers a non-specific and immediate response, while adaptive immunity is specific, develops over time, and offers long-lasting protection. Lysozyme is a non-specific component of the innate immune response known for its antimicrobial activity. In our study, we observed a significant increase in *lyz* expression levels only in PBLs of vaccinated fish. Additionally, *lyz* expression was not observed in challenged fish. These findings suggest that *lyz* may not play a primary role in the antiviral response, in accordance with previous research indicating its greater association with antibacterial rather than antiviral activity [22, 23]. Inflammatory cytokines such as IFN-γ and Mx, a member of the interferon-inducible gene, are essential components of innate immunity against viral infections in vertebrates [24]. Our study showed strong upregulation of *ifng* and *mx* early after both prime and booster vaccination, and in challenged fish. These findings support the well-known importance of the interferon pathway in SDDV elimination, as demonstrated in previous studies related to other viral infections and vaccine development [25–27]. In this study, another cytokine examined was IL-8, a chemokine known for its role in promoting inflammation and its association with autoimmune, inflammatory, and infectious diseases [28]. IL-8 is known to activate monocytes and macrophages, leading to the release of additional IL-8. This further facilitates the migration of various immune cells like neutrophils, basophils, and T lymphocytes to the site of infection [28]. The *il8* expression levels were upregulated shortly after both prime and booster vaccination in the head kidney and PBLs of vaccinated fish. Similarly, challenged fish exhibited elevated *il8* expression levels within 1–3 days after SDDV infection, underscoring the importance of IL-8 in combating SDDV. In a previous study on Olive flounder (*Paralichthys olivaceus*) infected with viral hemorrhagic septicemia virus (VHSV), it was noted that *il8* expression increased at the onset of the disease, highlighting the significant role of fish IL-8 in the antiviral immune response [29]. Furthermore, IL-8 has been implicated in regulating various immunological processes and promoting inflammatory responses in Nile tilapia infected with *S. agalactiae* and *A. hydrophila* [30]. Dendritic cells are recognized as professional antigen-presenting cells capable of capturing, processing, and presenting foreign antigens to T cells via major histocompatibility complex (MHC) molecules to initiate the adaptive immune response. In teleost fish, these cells can be derived in larger numbers from hematopoietic tissue and peripheral blood mononuclear cells [31]. Our study showed gene expression was associated with APCs (*dcst* and *mhc2a1*) in the head kidney earlier than PBLs. In the head kidney, these genes were upregulated within 7 dpv following both prime and booster vaccinations. However, in PBLs, upregulation of these genes occurred only after the booster vaccination (28 dpv), indicating a later response in PBLs. Previous research on rainbow trout (*Oncorhynchus mykiss*) reported co-expression of *mhc2* with *dcs*, suggesting their role in antigen presentation [31, 32]. A study by Frei et al. [33] highlighted the role of MHCII in activating toll-like receptors (TLRs) 2 and 4, contributing to the innate immune response.

Regarding adaptive immunity, T-lymphocyte markers *cd4* and *cd8* showed a delayed upregulation, appearing four weeks post-vaccination in both vaccinated and challenged fish. This suggests that the vaccine promotes a signaling pathway for the recognition and presentation of antigens, ultimately leading to the development of specific immune responses and memory against the virus. A similar pattern of upregulation was observed in Olive flounder vaccinated with an inactivated *Edwardsiella tarda* vaccine, where the expression levels of *mhc1a*, *mhc2a*, *cd4-1*, and *cd8a* were significantly higher in the vaccinated group compared to the control group after challenge with *E. tarda* [34]. For the humoral immune (HMI) response, they found that *ighm* exhibited late upregulate at 28 dpv. This finding did not correlate with the detection of serum and mucus IgM at 14 dpv.

To assess the vaccine efficacy of both prime and prime-booster vaccination methods, the vaccinated fish were challenged with SDDV at 42 dpv. Our results demonstrate a substantial increase in survival (66.67%) compared to the prime vaccination alone. This suggests that the prime-booster approach strengthens the fish’s immunological memory, helping the fish to produce rapid and long-term protection against the virus. In the study of de Groof et al. [2], three types of SDDV vaccines were developed for Asian seabass, including formalin-inactivated, binary ethyleneimine (BEI)-inactivated, and recombinant MCP (recMCP) vaccines. The vaccinated fish were then challenged with SDDV at 28 dpv, resulting in RPS of 74%, 70%, and 90% for formalin-, BEI-inactivated, and recMCP vaccines, respectively [2]. These RPS values were slightly higher than those observed for our SDDV-FIV compared to the same type of vaccine (whole virus particle inactivated). Another study introduced a formalin-killed bivalent vaccine comprising ISKNV-I and SDDV (strain ZH06/20) for Asian seabass [3]. It is important to highlight that the strain of SDDV employed was derived from yellow seabream rather than the Asian seabass, the species most recognized as vulnerable to SDDV. The vaccine efficacy was assessed through challenge tests against SDDV at 21 dpv, resulting in an RPS of 86.7% for vaccinated fish [3]. This percentage was higher than that observed for the SDDV-FIV in the present study and for the inactivated vaccines developed by [2]. In both studies, the timing of the challenge was earlier than our study, potentially leading to lower mortality rates. This time discrepancy could be attributed to the relatively high antibody titers and immune responses present at that time. Furthermore, their vaccine formulation incorporated oil adjuvants, which our vaccine lacked. Previous studies have demonstrated the efficacy of adjuvants in enhancing vaccine efficacy and immune response [18, 20, 21]. The study of de Groof et al. demonstrated the efficacy of a recombinant vaccine based on MCP protein which has previously been suggested that MCP is promising candidates for vaccine development in other members of the *Iridoviridae* family [2]. This highlights the potential benefit of exploring improved vaccine formulations incorporating immunostimulant agents, delivery platforms, or alternative vaccine types like the promising recombinant vaccine that offers remarkable protection against SDDV.

The clinical signs and gross lesions observed in SDDV-challenged fish were consistent with those documented in both experimental challenges and natural infections in previous studies [2, 7, 8]. These signs included darkening of the body, tail and fin erosion, broken fins, skin hemorrhages, scale loss, cloudy eyes, pale gills, liver hemorrhages, and enlarged spleens and kidneys. The pathological features were similar, and the disease progression pattern described by de Groof et al. was similar to our findings [2]. Specifically, the onset of SDD symptoms occurred around 5 days post-challenge, becoming clearly visible by 14 days post-infection, with mortality peaking at approximately 15–20 days post-infection [2]. Conversely, fish challenged with SDDV ZH06/20, originating from yellow seabream, exhibited distinct pathological characteristics, notably liver blood loss [3]. These findings suggest that the virulence of each SDDV strain may be host-dependent, with differing levels of virulence.

## Conclusions

Our study demonstrated the effectiveness of the SDDV FIV vaccine in protecting Asian seabass against SDDV infection. The vaccinated fish exhibited elevated serum and skin mucus antibodies (IgM), indicating a specific immune response against the virus. Additionally, we identified key genes associated with innate and adaptive immune responses. The observed upregulation of these genes in both the head kidney and PBLs at different time points suggests the vaccine’s ability to induce both rapid and long-term protection against SDDV. The key immune responses likely responsible for protective efficacy against the virus involve the induction of specific IgM antibodies and the activation of non-specific immune responses, such as activation of IFN-γ, IL-8, and Mx activities. Future studies should explore strategies such as optimizing vaccine formulation, incorporating adjuvants, and investigating alternative delivery platforms to further enhance the vaccine’s efficacy.

## Data Availability

All data generated or analysed during this study are included in this published article. Raw data are available from the corresponding author on reasonable request.

## Abbreviations

ELISA: Enzyme-linked immunosorbent assay
IgM: Immunoglobulin M
IP: Intraperitoneal
PBLs: Peripheral blood lymphocytes
SDD: Scale drop disease
SDDV: Scale drop disease virus
SDDV-FIV: Formalin-inactivated SDDV vaccine

## References

[1] Department of Fisheries (DOF) (2023). Estimation of aquatic production and value from fisheries in Thailand 2023–2025.

[2] de Groof A, Guelen L, Deijs M, van der Wal Y, Miyata M, Ng KS (2015). A Novel Virus causes Scale Drop Disease in *Lates calcarifer*. PLoS Pathog.

[3] Fu Y, Li Y, Zhang W, Fu W, Li W, Zhu Z, et al. Effectively protecting Asian seabass *lates calcarifer* from ISKNV-I, ISKNV-II, RSIV-II and SDDV by an inactivated ISKNV-I and SDDV bivalent vaccine. Aquaculture. 2023;566. 10.1016/j.aquaculture.2022.739218.

[4] Girisha SK, Puneeth TG, Nithin MS, Naveen Kumar BT, Ajay SK, Vinay TN (2020). Red sea bream iridovirus disease (RSIVD) outbreak in Asian seabass (*Lates calcarifer*) cultured in open estuarine cages along the west coast of India: first report. Aquaculture.

[5] Thanasaksiri K, Takano R, Fukuda K, Chaweepack T, Wongtavatchai J (2019). Identification of infectious spleen and kidney necrosis virus from farmed barramundi *lates calcarifer* in Thailand and study of its pathogenicity. Aquaculture.

[6] Tsai J-M, Huang S-L, Yang C-D. PCR Detection and Phylogenetic Analysis of Megalocytivirus isolates in Farmed Giant Sea Perch *Lates calcarifer* in Southern Taiwan. Viruses. 2020; 12(6). 10.3390/v12060681 PMC7354458 32599850

[7] Nurliyana M, Lukman B, Ina-Salwany MY, Zamri-Saad M, Annas S, Dong HT (2020). First evidence of scale drop disease virus in farmed Asian seabass (*Lates calcarifer*) in Malaysia. Aquaculture.

[8] Senapin S, Dong H, Meemetta W, Gangnonngiw W, Sangsuriya P, Vanichviriyakit R (2019). Mortality from scale drop disease in farmed *Lates calcarifer* in Southeast Asia. J Fish Dis.

[9] Ma J, Bruce TJ, Jones EM, Cain KD. A review of Fish Vaccine Development Strategies: conventional methods and modern biotechnological approaches. Microorganisms. 2019;7(11). 10.3390/microorganisms7110569 PMC6920890 31744151

[10] Ito T, Maeno Y (2015). Effect of booster shot and investigation of vaccination efficacy period against herpesviral haematopoietic necrosis (HVHN) in goldfish *Carassius auratus*. Vet Microbiol.

[11] Lopez-Vazquez C, Souto S, Olveira JG, Riaza A, Gonzalez O, Brea C et al. Nervous necrosis virus (NNV) booster vaccination increases Senegalese Sole Survival and enhances immunoprotection. Anim (Basel). 2022;13(1). 10.3390/ani13010051 PMC9817516 36611661

[12] Munang’andu HM, Evensen O (2019). Correlates of protective immunity for fish vaccines. Fish Shellfish Immunol.

[13] Sriisan S, Boonchird C, Thitamadee S, Sonthi M, Thanh Dong H, Senapin S (2020). A sensitive and specific SYBR Green-based qPCR assay for detecting scale drop disease virus (SDDV) in Asian sea bass. Dis Aquat Organ.

[14] Kurita J, Nakajima K, Hirono I, Aoki T (1998). Polymerase chain reaction (PCR) amplification of DNA of red sea bream iridovirus (RSIV). Fish dis res.

[15] Lugert V, Thaller G, Tetens J, Schulz C, Krieter J (2016). A review on fish growth calculation: multiple functions in fish production and their specific application. Rev Aquac.

[16] Livak KJ, Schmittgen TD (2001). Analysis of relative gene expression data using real-time quantitative PCR and the 2^–∆∆CT^ method. Methods.

[17] Fu Y, Li Y, Fu W, Su H, Zhang L, Huang C et al. Scale Drop Disease Virus Associated Yellowfin Seabream (*Acanthopagrus latus*) Ascites diseases, Zhuhai, Guangdong, Southern China: the first description. Viruses. 2021;13(8). 10.3390/v13081617 PMC8402775 34452481

[18] Monir MS, Yusoff MSM, Zulperi ZM, Hassim HA, Zamri-Saad M, Amal MNA (2021). Immuno-protective efficiency of feed-based whole-cell inactivated bivalent vaccine against *Streptococcus* and *Aeromonas* infections in red hybrid tilapia (*Oreochromis niloticus* x *Oreochromis mossambicus*). Fish Shellfish Immunol.

[19] Su L, Guo H, Guo B, Yi J, Yang Z, Zhou S et al. Efficacy of bivalent vaccine against *Aeromonas salmonicida* and *Edwardsiella tarda* infections in turbot. Fish Shellfish Immunol. 2023;139. 10.1016/j.fsi.2023.108837 37269913

[20] Sun F, Wu Y, Zhang Y, Liu Q, Wang Q, Liu X. An aluminium adjuvant compound with ginseng stem leaf saponins enhances the potency of inactivated *Pseudomonas plecoglossicida* vaccine in large yellow croaker (*Larimichthys crocea*). Fish Shellfish Immunol. 2024;144. 10.1016/j.fsi.2023.109243 37995892

[21] Zhang L-J, Chen Q, Yang J-X, Ge J-Q. Immune responses and protective efficacy of American eel (*Anguilla rostrata*) immunized with a formalin-inactivated vaccine against Anguillid Herpesvirus. Fish Shellfish Immunol. 2024;144. 10.1016/j.fsi.2023.109262 38040135

[22] Hikima J-i, Hirono I, Aoki T, Shimizu N, Aoki T, Hirono I, Takashima F (2003). The lysozyme gene in fish. Aquatic Genomics: steps toward a great future.

[23] Myrnes B, Seppola M, Johansen A, Øverbø K, Callewaert L, Vanderkelen L (2013). Enzyme characterisation and gene expression profiling of Atlantic salmon chicken- and goose-type lysozymes. Dev Comp Immunol.

[24] Secombes CJ, Belmonte R, Adam A (2016). Overview of the fish adaptive immune system.

[25] Jung MH, Jung SJ (2017). Protective immunity against rock bream iridovirus (RBIV) infection and TLR3-mediated type I interferon signaling pathway in rock bream (*Oplegnathus fasciatus*) following poly (I:C) administration. Fish Shellfish Immunol.

[26] Jung MH, Nikapitiya C, Kim SJ, Han HJ, Kim MS, Choi HS (2022). Protective immunity induced by ankyrin repeat-containing protein-based DNA vaccine against rock bream iridovirus (RBIV) in rock bream (*Oplegnathus fasciatus*). Virus Res.

[27] Poisa-Beiro L, Dios S, Montes A, Aranguren R, Figueras A, Novoa B (2008). Nodavirus increases the expression of Mx and inflammatory cytokines in fish brain. Mol Immunol.

[28] Matsushima K, Yang D, Oppenheim JJ (2022). Interleukin-8: an evolving chemokine. Cytokine.

[29] Kim KH, Kim HC, Park CJ, Park JW, Lee YM, Kim WJ (2019). Interleukin-8 (IL-8) expression in the Olive Flounder (*Paralichthys olivaceus*) against viral hemorrhagic septicemia virus (VHSV) challenge. Dev Reprod.

[30] Li X, Jiang B, Zhang Z, Huang M, Feng J, Huang Y (2023). Interleukin-8 involved in Nile Tilapia (*Oreochromis niloticus*) against bacterial infection. Fish Shellfish Immunol.

[31] Bassity E, Clark TG (2012). Functional identification of dendritic cells in the teleost model, rainbow trout (*Oncorhynchus mykiss*). PLoS ONE.

[32] Granja AG, Leal E, Pignatelli J, Castro R, Abos B, Kato G (2015). Identification of Teleost skin CD8alpha + dendritic-like cells, representing a potential common ancestor for mammalian cross-presenting dendritic cells. J Immunol.

[33] Frei R, Steinle J, Birchler T, Loeliger S, Roduit C, Steinhoff D (2010). MHC class II molecules enhance toll-like receptor mediated innate immune responses. PLoS ONE.

[34] Wu X, Xing J, Tang X, Sheng X, Chi H, Zhan W (2022). Protective cellular and humoral immune responses to *Edwardsiella tarda* in flounder (*Paralichthys olivaceus*) immunized by an inactivated vaccine. Mol Immunol.

[35] Meachasompop P, Bunnoy A, Keaswejjareansuk W, Dechbumroong P, Namdee K, Srisapoome P. Development of immersion and oral bivalent nanovaccines for streptococcosis and columnaris disease prevention in fry and fingerling Asian seabass (*Lates calcarifer*) nursery farms. Vaccines (Basel). 2023;12(1). 10.3390/vaccines12010017 PMC10818643 38250830

[36] Tumree P, Bunnoy A, Tang X, Srisapoome P (2024). Efficacy of whole-cell-based monovalent and bivalent vaccines against *Streptococcus iniae* and *Flavobacterium covae* in fingerling Asian seabass (*Lates calcarifer*). Fish Shellfish Immunol.

[37] Muangrerk C, Uchuwittayakul A, Srisapoome P. Identification, expression and antimicrobial functional analysis of interleukin-8 (IL-8) in response to *Streptococcus iniae* and *Flavobacterium covae* in Asian seabass (*Lates calcarifer* Bloch, 1790). Animals (Basel). 2024;14(3). 10.3390/ani14030475 PMC10854937 38338118

[38] Bunnoy A, Thangsunan P, Chokmangmeepisarn P, Yata T, Klongklaew N, Pirarat N, Kitiyodom S, Srisapoome S, Rodkhum C (2022). Mucoadhesive cationic lipid-based *Flavobacterium Oreochromis* nanoencapsulation enhanced the efficacy of mucoadhesive immersion vaccination against columnaris disease and strengthened immunity in Asian sea bass (*Lates calcarifer*). Fish Shellfish Immunol.

[39] Yang R, Han M, Fu Z, Wang Y, Zhao W, Yu G, Ma Z. Immune responses of Asian seabass *lates calcarifer* to dietary *Glycyrrhiza uralensis*. Anim (Basel). 2020;10(9). 10.3390/ani10091629 PMC7552140 32932808

[40] Saengrung J, Bunnoy A, Du X, Huang L, An R, Liang X, Srisapoome P (2023). Effects of ribonucleotide supplementation in modulating the growth of probiotic *Bacillus subtilis* and the synergistic benefits for improving the health performance of Asian seabass (*Lates calcarifer*). Fish Shellfish Immunol.

